# Demonstration of three-dimensional contact point determination and contour reconstruction during active whisking behavior of an awake rat

**DOI:** 10.1371/journal.pcbi.1007763

**Published:** 2022-09-15

**Authors:** Lucie A. Huet, Hannah M. Emnett, Mitra J. Z. Hartmann

**Affiliations:** 1 Department of Mechanical Engineering, Northwestern University, Evanston, Illinois, United States of America; 2 Department of Biomedical Engineering, Northwestern University, Evanston, Illinois, United States of America; University of Colorado Boulder, UNITED STATES

## Abstract

The rodent vibrissal (whisker) system has been studied for decades as a model of active touch sensing. There are no sensors along the length of a whisker; all sensing occurs at the whisker base. Therefore, a large open question in many neuroscience studies is how an animal could estimate the three-dimensional (3D) location at which a whisker makes contact with an object. In the present work we simulated the shape of a real rat whisker to demonstrate the existence of several unique mappings from triplets of mechanical signals at the whisker base to the three-dimensional whisker-object contact point. We then used high speed video to record whisker deflections as an awake rat whisked against a peg, and used the mechanics resulting from those deflections to extract the contact points along the peg surface. These results demonstrate that measurement of specific mechanical triplets at the base of a biological whisker can enable 3D contact point determination during natural whisking behavior. The approach is viable even though the biological whisker has non-ideal, non-planar curvature, and even given the rat’s real-world choices of whisking parameters. Visual intuition for the quality of the approach is provided in a video that shows the contour of the peg gradually emerging during active whisking behavior.

## Author summary

When a rodent explores its environment, it often rhythmically taps its whiskers against objects to obtain tactile (touch) information. This behavior is called “whisking.” In the field of neuroscience, whisking is used to investigate how animals combine tactile information with movement to explore objects. Like a hair, a whisker has no sensors along its length–all sensors are at the whisker base. So how do a rat’s whiskers convey information about the three-dimensional (3D) location of an object to the rat’s brain? When a whisker touches an object, six mechanical signals are generated at the whisker base. Previous work has shown that only three of these six mechanical signals are required to determine 3D contact location, but this previous work was performed only in simulation, using idealized deflections of idealized whiskers. Here we identify mappings between mechanical signals and 3D object location during active whisking of a real rat. We then use one mapping to show a sequence of whisker-object contact points that gradually reveals the contour of the object. This work shows that at least one triplet of mechanical signals is sufficient to extract 3D contours through touch, even though real-world whisking includes friction, dynamics, and non-idealized whisker geometries.

## Introduction

Rats and mice can obtain detailed tactile information by rhythmically sweeping their whiskers back and forth against surfaces and objects in the environment, a behavior called “whisking.” They can use this whisker-based tactile information to determine an object’s location, size, orientation, and texture [1–6]. How rats achieve these tasks is still an open question, especially given that a whisker is simply a cantilever beam with no sensors along its length. Numerous neurophysiological and behavioral studies have specifically investigated how a rodent might use a single whisker to determine the location of a vertical peg [5,7–21]. Studies have shown that although barrel cortex is required for peg localization [13], knowledge of instantaneous whisker position is not [14]. To date, however, studies have not been able to determine the exact physical cues that the animal might use for peg localization, in part because they have been limited to a two-dimensional (2D) analysis of whisker motion and object contact, with the third dimension sometimes attributed to whisker identity [8,9]. In addition, nearly all of these studies have examined localization in head-centered coordinates, which can involve cues such as the time of contact relative to the start of a whisk, or an efference copy containing information about the midpoint of the whisk [21]. The physical cues that contain information about 3D object location in whisker-centered coordinates have not been established. Whisker-centered coordinates are defined with respect to the basepoint and reference frame of each individual whisker [22], and they are the coordinate system in which tactile information is most directly conveyed to mechanoreceptors in the whisker follicle [22–25].

Complementing the biological literature, several studies in the field of robotics have investigated the problem of whisker-object contact point determination in three dimensions [26–29]. Similar to the present work, these studies have focused on the use of quasistatic mechanical signals—three reaction forces and three moments at the whisker base—to infer the three-dimensional (3D) whisker-object contact point. Early work showed that using all six signals at the base of a stiff antenna was sufficient to determine the 3D contact point location [27,29], and more recent work in simulation has shown that in many cases only three of the six mechanical signals are actually required [28]. These simulations have indicated that the particular mechanical “triplets” sufficient for 3D contact point determination depend on the intrinsic shape of the whisker, that is, whether it is cylindrical or tapered, straight or curved [28]. From a computational perspective, triplets of mechanical signals are important because they represent the theoretical “minimum set” necessary to represent a 3D contact point. Examining which particular mechanical triplets are associated with uniquely invertible mappings to the 3D contact point can yield insights into mechanical redundancies and thus approaches towards dimensionality reduction.

The present work brings together the fields of biology and robotics to ask how the mechanics associated with whisker bending could allow an actively whisking rat to determine the 3D location of whisker-object contact. Biological whiskers do not have an idealized geometry, and the distal segment of a whisker often curves out of the plane established by its proximal portion [25, 30–32]. In addition, active whisking behavior often generates collisions that can contain significant dynamic effects. It is therefore not at all clear that the results of idealized simulation work will generalize to the case of a biological whisker as used by a rodent during real whisking behavior. There is no guarantee that the mapping from 3D contact point to mechanical signals will be uniquely invertible for a biological whisker, using three or even four mechanical signals.

The goal of the present work was to determine which “minimal sets”—if any—of mechanical signals at the base of a biological whisker could generate a unique mapping to the 3D contact point, during real whisking behavior involving real whisker-object contact. We used high speed video to record 3D deflections of the “gamma” or “γ” whisker as an awake rat whisked against a peg, and then simulated the mechanical signals generated by a whisker of that exact shape. We first show that there exist several unique mappings from triplets of mechanical signals at the whisker base to the 3D location of the whisker-object contact point. We then select one of these mappings to show that it can be used to extract the 3D contact points, and thus the contour, of a peg placed in front of the animal. Results are discussed in terms of their implications for dimensionality reduction in the follicle, and are expected to generalize across all biologically-realistic whisker shapes with a few exceptions, addressed in the discussion.

## Results

### Problem statement: mapping mechanical signals at the whisker base to the 3D whisker-object contact point location

**Fig 1:**
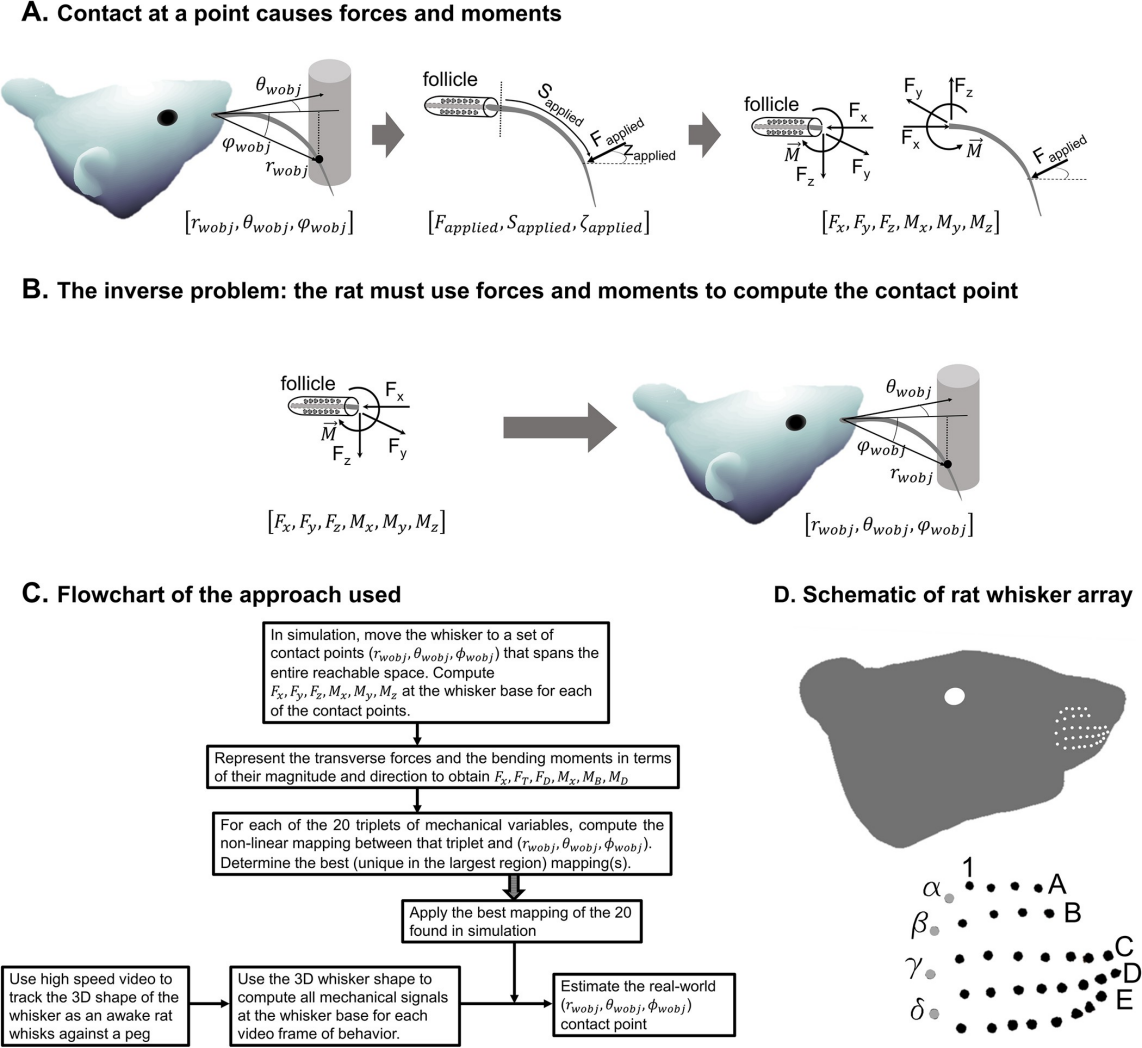
When a rat whisks against an object, forces and moments are generated at the whisker base; the rat must use these forces and moments to determine the 3D contact point location. **(A)** The 3D contact point between the whisker and the object is denoted as r_wobj_, θ_wobj_, and φ_wobj_. This contact point exerts a force on the whisker (F_applied_), which generates reaction forces and moments at the whisker base. **(B)** The inverse problem: the rat’s nervous system must perform the inverse of the process depicted in (A). **(C)** A flowchart that describes the approach taken to determine the non-linear mappings that could allow the rat to solve this inverse problem. The 20 mappings indicated in the center box are listed in Fig A in S1 Text. (D) A schematic of a rat head and approximate locations of the whisker basepoints. Whiskers in the caudal-most column (denoted as gray dots in the expanded view) are identified with the Greek letters α through δ. Columns are numbered from caudal to rostral, and rows are lettered from dorsal to ventral.

When a rat whisks against an object, as depicted in Fig 1A, the contact point between the whisker and the object is denoted by the coordinates (r_wobj_, θ_wobj_, φ_wobj_) relative to the whisker basepoint, where the subscript “wobj” stands for whisker-object [22]. The whisker’s deflection causes reaction forces and moments (torques) at the whisker base, denoted as *F*_*x*_, *F*_*y*_, *F*_*z*_, *M*_*x*_, *M*_*y*_, and *M*_*z*_. The force *F*_*x*_ is called the “axial” force because it acts directly along the whisker’s long axis at the whisker base. The axial force is positive when it pulls the whisker directly out of the follicle and negative when it pushes the whisker directly into the follicle. The forces *F*_*y*_ and *F*_*z*_ are called “transverse” forces, because they act perpendicular (“transversely”) to the whisker at the whisker base. *M*_*x*_ is called the “twisting” moment because it twists the whisker about its long axis, while *M*_*y*_ and *M*_*z*_ are called the “bending” moments because they cause the whisker to bend.

During exploratory behavior, the rat must solve the inverse problem, illustrated in Fig 1B: it must use the mechanical signals at the whisker base to determine the 3D contact point location. Under the assumption of quasistatic contact, it can be theoretically shown that the six mechanical signals, *F*_*x*_, *F*_*y*_, *F*_*z*_, *M*_*x*_, *M*_*y*_, and *M*_*z*_ are always sufficient to uniquely determine the 3D contact point location (r_wobj_, θ_wobj_, φ_wobj_) [27,29]. Simulation work has shown that for the case of a planar, perfectly tapered whisker with parabolic curvature, some “triplet” combinations of these six mechanical signals are also sufficient.

These theoretical and simulation results, however, leave several important questions unanswered. First, for a non-idealized, biological whisker, are all six mechanical signals really needed to determine the 3D contact point location, or—as is the case for the idealized whisker–might only a subset of them (e.g., a triplet) be sufficient? Second, assuming that uniquely invertible “minimal set” mappings were found, what is the nature of these mappings, and could they be used during real-world active whisking to determine the contours of an object? Taken together with our knowledge of mechanoreceptor distribution within the follicle [33–35], the answers to these questions may help constrain the neural computations that could permit object localization (see *Discussion*).

The procedure used to answer these questions is depicted in the flowchart of Fig 1C. The 3D shape of a real whisker is obtained, and the whisker is then simulated to be deflected to a gridded sampling of contact points across its entire reachable space (see *Methods*). For each deflection, the forces and moments at the base of the whisker are computed, and, for convenience, the transverse forces and the bending moments are rewritten in terms of their magnitude and direction. An initial analysis of the data indicated that the smallest “minimal set” that could generate a uniquely invertible mapping was a triplet. Therefore, each possible triplet of the six mechanical variables is investigated to determine in which regions it yields a unique mapping to the (r_wobj_, θ_wobj_, φ_wobj_) contact point. The best mapping (i.e., the one that is unique in the largest region) is selected.

In parallel, high speed video is used in behavioral experiments to obtain the 3D shape of the “gamma” (γ) whisker as an awake rat whisks against a peg. For each video frame, the 3D whisker shape is used to compute the forces and moments at the whisker base. The best mapping—obtained from the simulation steps described above (again, with “best” defined as the one that has the largest region of uniqueness)—is then applied in each video frame to obtain an estimate of the 3D contact point for that frame.

### Simulating forces and moments at the base of whisker deflected to all possible positions

**Fig 2:**
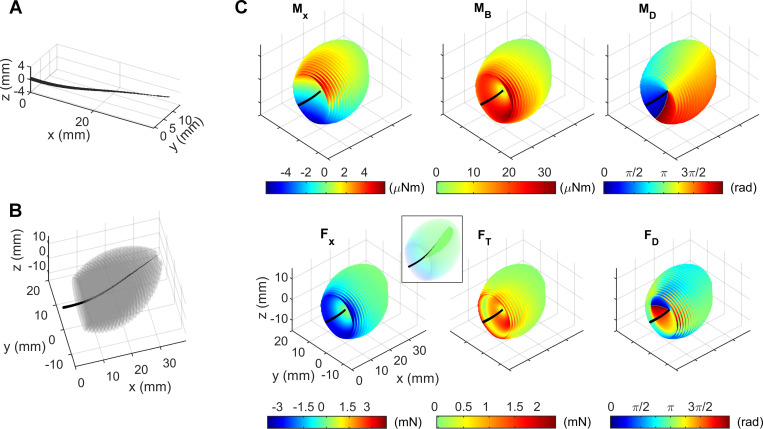
Forces and moments at the whisker base for all possible contact points that the whisker can reach. **(A)** The black line represents the shape of the whisker with its base placed at the origin. The taper in the whisker is only for visual clarity and is not to scale. **(B)** The contact points that the whisker was able to reach are plotted as a series of translucent gray surfaces; each surface represents a set of reachable contact points at a single radial distance. The translucent surfaces are so close together that they merge into a cloud of points in the shape of an irregular ellipsoid. **(C)** The same whisker and point cloud as in **(B)** are shown, with the contact points now colored according to the mechanical variable indicated in each panel. The plots in all six panels have the same axes as the plot for *F*_*x*_. The inset in the bottom left panel plots negative values of *F*_*x*_ as transparent and positive values as opaque in order to reveal the small region of positive *F*_*x*_ values surrounding the whisker (intense green).

As shown in Fig 1D, approximately 30 whiskers are arranged in regular rows and columns on the rat’s face; the rows are identified with the letters A–E and the columns with the numbers 1–7. The caudal most column contains only four whiskers, identified by the Greek letters α through δ. Following the procedure depicted in Fig 1C, we began by characterizing the 3D shape of the “gamma” (γ) whisker, as illustrated in Fig 2A. The γ whisker was chosen because it has an imperfect geometry (it is not an idealized parabola [25,30,31] or Euler spiral [32,36] and has significant out of plane curvature). We then simulated deflecting the whisker to a gridded sampling of the 3D point locations it could reach and computed the resulting forces and moments at the whisker base. The set of reachable contact points is depicted as a gray point cloud about the whisker in Fig 2B. For each gray point in Fig 2B, we computed the six forces and moments at the whisker base (*F*_*x*_, *F*_*y*_, *F*_*z*_, *M*_*x*_, *M*_*y*_, *M*_*z*_). We then decomposed the transverse forces and the bending moments into their magnitude and direction:

$$Magnitude\;of\;the\;transverse\;force:\;F_{T}=\sqrt{F_{y}^{2}+F_{z}^{2}}$$ (1)

$$Direction\;of\;the\;transverse\;force:\;F_{D}=atan\;\left(\frac{F_{z}}{F_{y}}\right)$$ (2)

$$Magnitude\;of\;the\;bending\;moment:\;M_{B}=\sqrt{M_{y}^{2}+M_{z}^{2}}$$ (3)

$$Direction\;of\;the\;bending\;moment:\;M_{D}=atan\;\left(\frac{M_{z}}{M_{y}}\right)$$ (4)

These six signals, *F*_*x*_, *F*_*T*_, *F*_*D*_, *M*_*x*_, *M*_*B*_, and *M*_*D*_, are plotted in the six panels of Fig 2C. Each panel of Fig 2C shows the same black whisker and the same cloud of points as in Fig 2B, but viewed from a different angle for visual clarity. The contact points are colored according to the magnitude of the mechanical signal depicted in the panel. These colored ellipsoid-shaped plots reveal several important trends in the forces and moments at the whisker base.

Two notable trends can be observed in Fig 2C. Three mechanical signals, *F*_*x*_, *F*_*T*_, and *M*_*B*_, all exhibit the largest magnitude for proximal contacts (regardless of deflection magnitude) as well as for large angles of deflection (regardless of contact location). In addition, the signals *F*_*D*_ and *M*_*D*_ are ~90° offset from each other because each reaction moment occurs in a plane perpendicular to the reaction force.

The axial force, *F*_*x*_, has a small region of positive values; in these regions the axial force is pulling the whisker out of the follicle instead of pushing it in. These values occur for distal contacts that are concave forward with small deflections, i.e., the region in which the contact “straightens out” the whisker. This region is visible in the inset for *F*_*x*_ in Fig 2C, in which all negative *F*_*x*_ values are transparent and all positive values are opaque. The positive values are thus visible as the dark green area surrounding the whisker.

The transverse force, *F*_*T*_, generally follows the same trends as *M*_*B*_ and *F*_*x*_, but it also has a hollow “tube” of zero magnitude surrounding the whisker; the “ring” of its bottom end is visible in same relative region where *F*_*x*_ has a large magnitude negative value. This tube occurs when the whisker is bent such that the portion of the whisker local to the contact point is parallel to the y-z plane; in this case the force points entirely in the negative x-direction, resulting in zero *F*_*T*_. Contact points that deflect the whisker beyond this tube are defined as “large deflection” contacts. This flip into large deflections can also be seen by the sudden 180° change in *F*_*D*_.

The twisting moment, *M*_*x*_, exhibits very different trends from the other forces and moments. The magnitude primarily varies in the z-direction rather than radially. This effect occurs because the whisker exhibits more “twist” and therefore greater *M*_*x*_ magnitude as it is deflected out of the x-y plane.

Overall, these trends in forces and moments are similar, but not identical, to those for an idealized, planar, tapered whisker with a parabolic shape [28]. We therefore anticipated that we would see similar results for uniqueness of the mapping between subsets of the variables *F*_*x*_, *F*_*T*_, *F*_*D*_, *M*_*x*_, *M*_*B*_, *M*_*D*_ and *r*_*wobj*_, *θ*_*wobj*_, φ_*wobj*_.

### Unique mappings from triplets of mechanical variables to the 3D whisker-object contact point

Continuing to follow the procedure depicted in the flowchart of Fig 1C, we selected all possible triplets of mechanical signals and tested whether each triplet was sufficient to uniquely determine the 3D location of the whisker-object contact point, *r*_*wobj*_, *θ*_*wobj*_, φ_*wobj*_. Three triplets of mechanical variables were found to meet the criteria for uniqueness: (*F*_*x*_, *M*_*B*_, *M*_*D*_), (*M*_*x*_, *M*_*B*_, *M*_*D*_), and (*F*_*x*_, *F*_*D*_, *M*_*D*_). Criteria for uniqueness can be found in Section 4 in S1 Text. A list of the regions of non-uniqueness for the remaining 19 mappings is in Table A in S1 Text.

**Fig 3:**
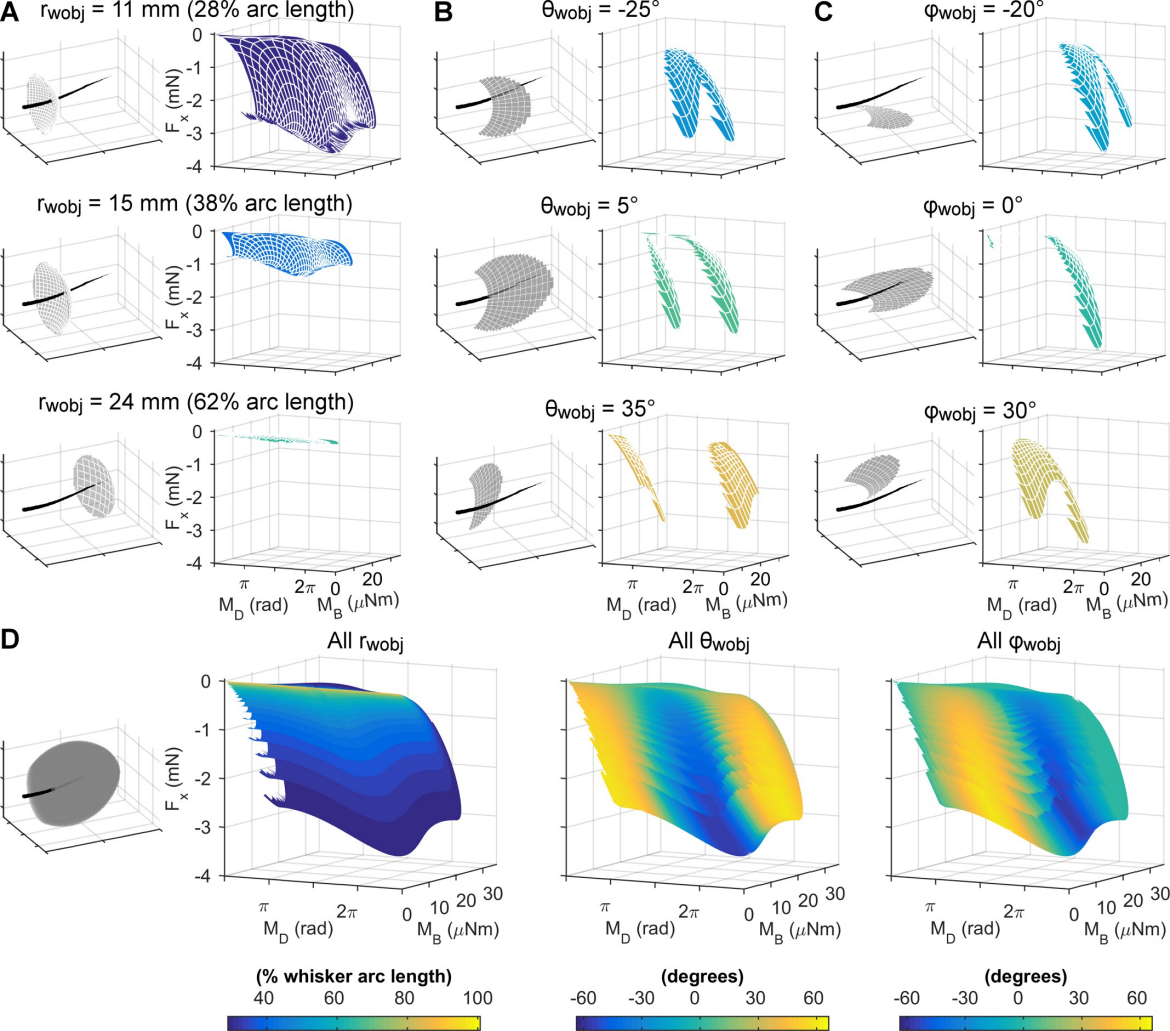
The mappings between mechanical variables and the 3D whisker-object contact point can be represented as solids, each drawn as series of monochromatic surfaces. The left columns of panels **A**, **B**, and **C** depict the whisker in black, with all reachable contact points shown as gray surfaces for the indicated radial distance (r_wobj_), azimuthal angle (θ_wobj_), or elevation angle (φ_wobj_). The right columns of panels **A**, **B**, and **C** plot the monochromatic surfaces that correspond to the F_x_, M_B_, and M_D_ signals for the indicated radial distances and angles. The colormaps for panels in **A**, **B**, and **C** are obtained from the colorbars in the corresponding columns of **D**. **(D)** The monochromatic surfaces for each of the r_wobj_, θ_wobj_, and φ_wobj_ mappings are layered together to form three “solids.” *First column*: The whisker is shown in black and all reachable contact points are shown in gray. This panel contains the identical points as Fig 2B. *Second*, *third*, *and fourth columns*: All the monochromatic surfaces for the different values of r_wobj_, θ_wobj_, and φ_wobj_ are plotted together in F_x_, M_B_, and M_D_ space. The “feathered edge” apparent in the second column is an artifact of the discretization of the r_wobj_ values.

Each of these successful mappings can be visualized with a set of three colored, solid shapes. However, these visualizations are unintuitive and challenging to understand. To provide intuition for how to visualize a mapping we show an example using the (*F*_*x*_, *M*_*B*_, *M*_*D*_) triplet. Fig 3 depicts the gradual construction of the three solids that represent the mapping between (*F*_*x*_, *M*_*B*_, *M*_*D*_) and (*r*_*wobj*_, *θ*_*wobj*_, φ_*wobj*_).

The three panels in the left column of Fig 3A show the whisker in black along with all the contact points it could reach at three different radial distances: 11 mm, 15 mm, and 24 mm. These distances correspond to 28%, 38% and 62% of the whisker arc length, respectively.

The three panels in the right column of Fig 3A show *F*_*X*_, *M*_*B*_, and *M*_*D*_ computed from the contact points at the three different radial distances. In each of these three right panels, the points representing *F*_*x*_, *M*_*B*_, and *M*_*D*_ are connected to form a single continuous surface. Each surface is monochromatic, indicating that all points within that surface are generated from contact points at the same radial distance. The surface corresponding to r_wobj_ = 15 mm is a different color from the surface corresponding to r_wobj_ = 11 mm, and its shape is “shrunk down” on the *F*_*x*_ and *M*_*B*_ axes. Similarly, the surface for *F*_*x*_, *M*_*B*_, and *M*_*D*_ corresponding to r_wobj_ = 24 mm is yet a third color and its shape has shrunk even more in *F*_*x*_ and *M*_*B*_.

Fig 3B shows a similar set of plots as Fig 3A, except that each surface represents a single θ_wobj_ angle, instead of a single value for r_wobj_. The three panels in the left column show gray contact points at three different values of θ_wobj_: -25°, 5°, and 35°, and the three panels in the right column show the corresponding monochromatic surfaces in the *F*_*x*_, *M*_*B*_, *M*_*D*_ space. Notice that the color scale for θ_wobj_ (Fig 3B) is independent of the color scale for r_wobj_ (Fig 3A).

Fig 3C shows similar plots for three different values of φ_wobj_: -20°, 0°, and 30°, with corresponding monochromatic surfaces in the *F*_*x*_, *M*_*B*_, *M*_*D*_ space. Again, notice that the color scale for φ_wobj_ is independent of the color scale for θ_wobj_ and r_wobj_.

Finally, Fig 3D combines all the surfaces for each geometric coordinate into its own plot. In the left column of Fig 3D all the contact points plotted as a gray cloud about the whisker; this figure matches Fig 2B. The second column in Fig 3D shows all the monochromatic surfaces in *F*_*x*_, *M*_*B*_, *M*_*D*_ space for r_wobj_. Notice that all the different surfaces are nested one inside the other. This set of surfaces represents the mapping “solid,” forming a lookup table to determine r_wobj_. If given three values, *F*_*x*_, *M*_*B*_, and *M*_*D*_ at the base of the whisker for an unknown contact, the color of the solid at that *F*_*X*_, *M*_*B*_, *M*_*D*_ location determines the radial distance. The solid lookup tables for *θ*_*wobj*_, and φ_*wobj*_ are shown in the third and fourth columns of Fig 3D; again, these solids are formed by plotting a series of monochromatic surfaces.

As expected, the solids in columns 2 through 4 of Fig 3D all have the same shape; they differ only in coloring. The “feathered edges” most noticeable on the visualization for r_wobj_ are a discretization artifact and do not have any significance. Using a larger number of values for r_wobj_ would cause the feathered edges to coalesce into the identical shape as those for *θ*_*wobj*_, and φ_*wobj*_.

Close visual examination of the solids in Fig 3D reveals an important feature: none of the monochromatic surfaces within any of the solids overlap. The absence of overlap indicates that a single reading of *F*_*x*_, *M*_*B*_, and *M*_*D*_ results in unique values for r_wobj_, *θ*_*wobj*_, and φ_*wobj*_, meaning that the mapping is unique. If any of the monochromatic surfaces overlapped or intersected, then the readings of *F*_*x*_, *M*_*B*_, and *M*_*D*_ at that point of intersection would result in multiple combinations of r_wobj_, *θ*_*wobj*_, and φ_*wobj*_, rendering the mapping non-unique.

Importantly, however, due to human fallibility, visual inspection is necessary but not sufficient to determine if a particular mapping is unique. In addition to visual inspection, we tested uniqueness using neural networks as non-linear function solvers (details provided in Section 4 in S1 Text). If a neural network could solve for a mapping, then the mapping was unique. Specifically, given (F_x_, M_B_, M_D_) as inputs and (r_wobj_, *θ*_*wobj*_, φ_*wobj*_) as outputs, the network had to be able to solve for the non-linear function that maps between inputs and outputs with sufficiently small errors. In other words, the neural network effectively generates a “look-up table” for the mapping, and the look-up table must be unique.

We found that–as expected–the look up table generated using (F_x_, M_B_, M_D_) as inputs contained especially high errors in r_wobj_ when the contact point was near the whisker and deflections were very small. These high errors are expected because tiny variations in the forces generated during small angle deflections can cause large changes in r_wobj_ for the estimated contact point. The look-up table did not have sufficient resolution to resolve contact points for these small angle deflections. In future work this issue could be addressed in part by changing the mesh distribution of the contact points, but in the present work we simply excluded plotting contact points that fell within a thin “cone” surrounding the whisker; exclusion criteria are described within the figure captions.

### Contact point determination in the awake, behaving rat

The mappings described in the previous sections were obtained purely from simulation. The simulations assumed that the whisker was rigidly clamped at its base and underwent ideal, frictionless point-deflections.

It is not at all evident that the mappings obtained from simulation will apply during active whisking behavior of an awake rat. The mapping results shown in Fig 3D could be degraded by many nonlinear effects, including tissue compliance [11], dynamics associated with inertia or collision with the peg, and non-ideal multi-point or sliding contact [37]. The mappings are therefore only of theoretical interest unless the associated lookup tables can be successfully applied to real-world whisking behavior.

**Fig 4:**
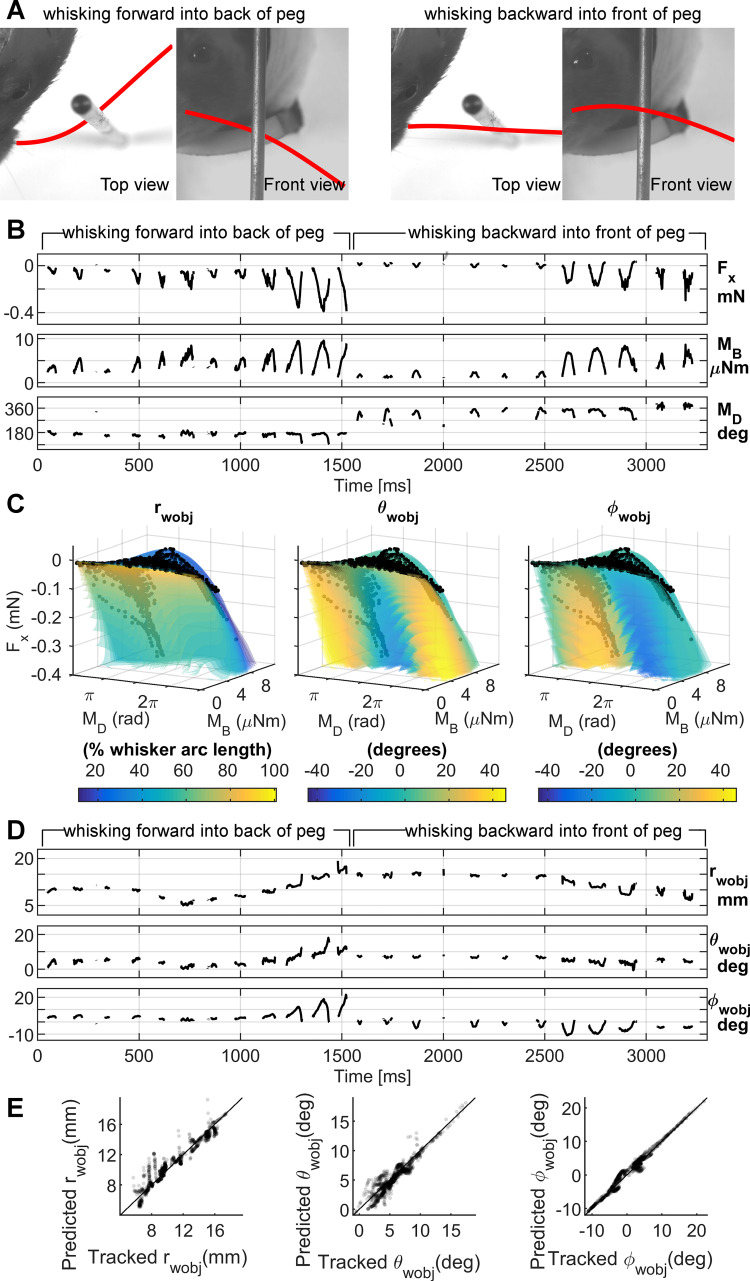
The 3D contact point (r_wobj_, θ_wobj_, φ_wobj_) can be determined from F_x_, M_B_, M_D_ during active whisking. The data in this figure exclude small angle deflections, i.e., any point that lay less than s_closest_*tan(2°) from the whisker, where s_closest_ is the arc length of the point on the whisker closest to the contact point. **(A)** Top and front views from the raw video data of a rat whisking forward into the back of the peg (left two images) and backwards into the front of the peg (right two images). The tracked whisker is traced in red. **(B)** The F_x_, M_B_, and M_D_ data output by the model as the rat whisked into the peg. Note the sudden change in M_D_ when the rat switches from whisking forward to whisking backward into the peg. **(C)** F_x_, M_B_, and M_D_ from **B** plotted onto the mapping visualizations for r_wobj_, θ_wobj_, and φ_wobj_. These visualizations show the relevant parts of the mappings from Fig 3D, and the monochromatic layers are plotted as translucent for better visualization. The black dots represent the data from **B** plotted onto F_x_, M_B_, M_D_ space. The color of the “solid” at the black dots in each of the three plots gives the contact point location coordinates: r_wobj_, *θ*_*wobj*_, φ_*wobj*_. **(D)** The r_wobj_, θ_wobj_, and φ_wobj_ values given by the mappings in **C (E)** Tracked values of r_wobj_, θ_wobj_, and φ_wobj_ plotted against the values of r_wobj_, θ_wobj_, and φ_wobj_ as predicted from the mechanical signals. The dots are translucent to improve visualization. The black diagonal line represents where the dots would lie if the 3D contact points computed from the mechanical signals were perfect.

To address this important concern, we recorded ~3.5 seconds of high-speed video as an awake rat whisked against a vertical peg (2.7 mm diameter). During this particular trial of whisking, the rat first whisked forward against the back of the peg. The whisker then slipped past the peg, and the rat whisked backwards against the front of the peg. Fig 4A shows the top and front views of the rat whisking forward and backward against the peg with the tracked whisker traced in red.

For each frame of video, we used the tracked 3D shape of the whisker and the tracked location of the 3D whisker-object contact point to compute the mechanical signals *F*_*x*_, *M*_*B*_, and *M*_*D*_ at the whisker base. These mechanical signals, shown in Fig 4B, represent the information we assume the rat has during whisking behavior [22,38,39]. Details for finding the 3D whisker shape and contact point location are provided in Fig B in S1 Text.

The three mechanical signals, *F*_*x*_, *M*_*B*_, and *M*_*D*_, were then used as the inputs to the mapping established in Fig 3D. During this particular trial of whisking, the signals spanned a more limited range than that shown in Fig 3D. Therefore, Fig 4C illustrates only the region of the mappings relevant to this particular whisking trial. Each of the mapping solids in Fig 4C is slightly translucent, so as to show a set of black dots representing the trajectory of the whisker through the F_x_, M_B_, and M_D_ space. This time-varying trajectory is best observed in S1 Video.

In Fig 4C, the color of each of the solid objects at the location of the black points yields the reading for the estimated contact point location. Using the appropriate look-up table for this mapping, we can obtain predicted values for the contact point (r_wobj_, *θ*_*wobj*_, φ_*wobj*_) which are plotted as functions of time in Fig 4D.

The quality of the predicted values for (r_wobj_, *θ*_*wobj*_, φ_*wobj*_) was evaluated by plotting them against the ground-truth tracked values for (r_wobj_, *θ*_*wobj*_, φ_*wobj*_); results are shown in Fig 4E. Data points for each measurement at 1 msec intervals are represented by a black dot. If they fall on the black diagonal line, the predicted value exactly matches the tracked value. The fact that the points generally lie close to the diagonal line shows the high quality of the mapping between mechanical signals and the 3D contact point.

As expected, predictions from the look-up table show the greatest errors for the variable r_wobj_. These errors result from limitations in the resolution of the mappings for small deflections: very small changes in force can cause large jumps in predictions for r_wobj_.

### Sensitivity analysis of the mapping between (*M*_*B*_, *M*_*D*_, *F*_*x*_) and (*r*_*wobj*_, *θ*_*wobj*_, *φ*_*wobj*_)

To quantify where the mapping between mechanical variables and contact points was most susceptible to small deviations in mechanical estimates, we calculated the Jacobian that relates contact point space and mechanical variable space. For our mapping, the Jacobian with respect to the contact point is the 3×3 matrix shown in Eq 5. Each term is a partial derivative of a contact point coordinate with respect to a mechanical signal. The subscript “wobj” on contact point coordinates is dropped for brevity.

In Eq 5, the top row describes how changes in mechanical variable measurement affect *r*_*wobj*_, the radial distance along the whisker length. Similarly, the middle and bottom rows describe how small changes in mechanical variable measurements will affect *θ*_*wobj*_ and *φ*_*wobj*_, the angles at which the whisker makes object contact. Thus, each column of the Jacobian shows how a single mechanical variable will affect the resultant contact point, and each row shows how all mechanical variables will individually affect a single contact point coordinate.


$${Jacobian}_{wobj}=\left[\begin{array}{ccc} \frac{\partial r}{{\partial M}_{D}} & \frac{\partial r}{{\partial M}_{B}} & \frac{\partial r}{{\partial F}_{x}}\\ \frac{\partial \theta }{{\partial M}_{D}} & \frac{\partial \theta }{{\partial M}_{B}} & \frac{\partial \theta }{{\partial F}_{x}}\\ \frac{\partial \varphi }{{\partial M}_{D}} & \frac{\partial \varphi }{{\partial M}_{B}} & \frac{\partial \varphi }{{\partial F}_{x}} \end{array}\right]$$ (5)

Because we do not have analytic functions defining the relationship between (*M*_*D*_, *M*_*B*_, *F*_*x*_) and (*r*_*wobj*_, *θ*_*wobj*_, *φ*_*wobj*_), we evaluated each element of the Jacobian numerically. Details of the approach are described in *Methods*. It is important to note that the sensitivity analysis was not run on the full simulation space shown in Fig 3, but rather on the range shown in Fig 4C which bounds the behavioral data. This choice allowed us to evaluate the mapping sensitivity in behaviorally-realistic ranges. A separate analysis in which the Jacobian was evaluated over the entire simulation range yielded similar results to those that follow.

**Fig 5:**
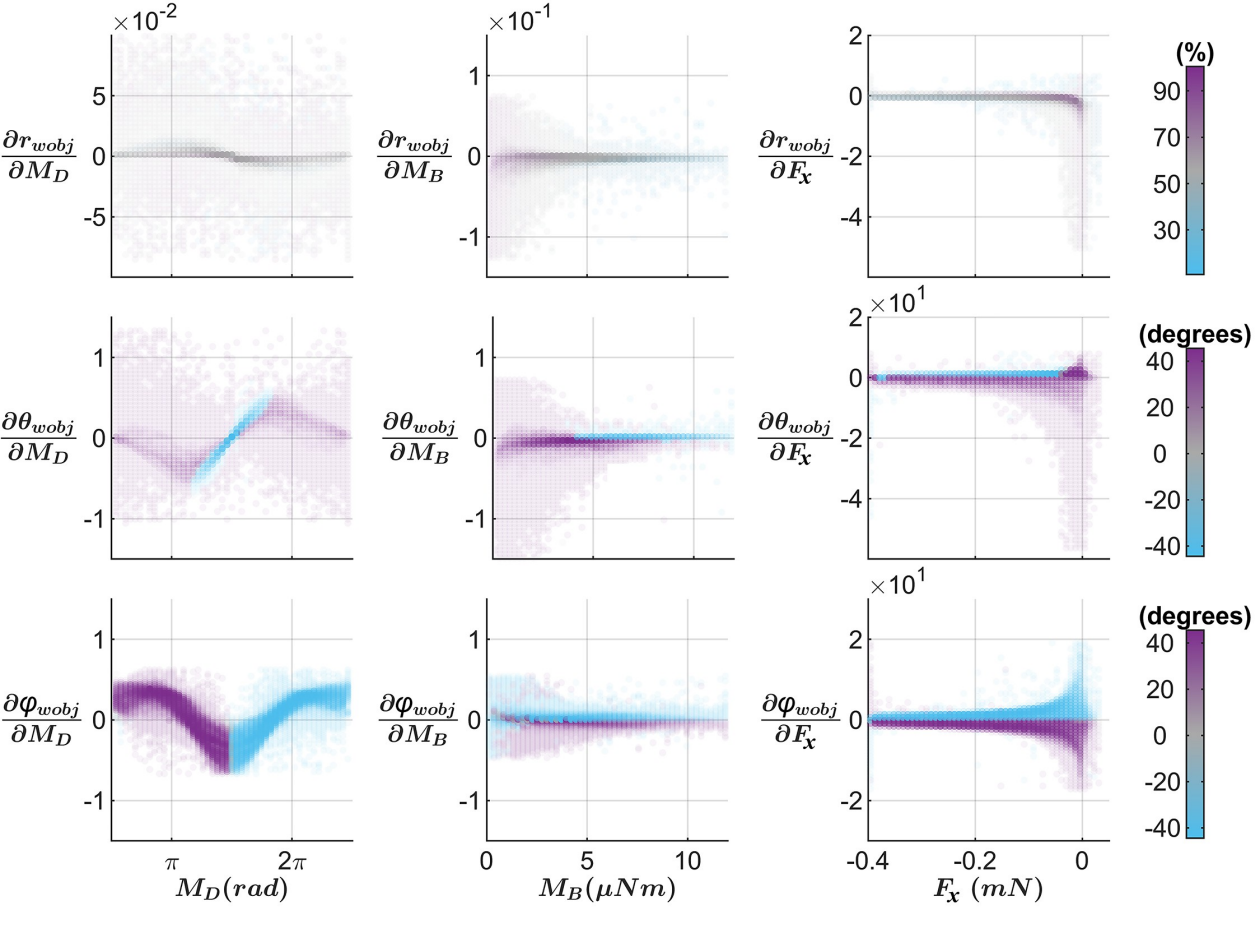
The mapping between contact point coordinates and mechancial variables is most sensitive at small values of *F*_*X*_ and *M*_*B*_. This figure illustrates how sensitive the contact point estimation is to small changes in mechanical variables. Each panel plots a single Jacobian element from Eq 5 against its corresponding mechanical variable, with the colormap determined by its corresponding contact point coordinate. Thus, all panels in a single column share the same x-axis and all panels in a single row share the same color map. From top to bottom, the colormaps indicate percent of whisker arclength, degrees in *θ*_*wobj*_, and degrees in *φ*_*wobj*_.

Fig 5 shows the results of the sensitivity analysis. Each panel shows a single Jacobian element plotted against its related mechanical variable, thus each column has the same x-axis. The colormap for each panel is determined by the contact point variable in the numerator, thus each row has the same color map. Because the units for each mechanical variable are different, we cannot compare the magnitudes of the Jacobian elements to each other. We can, however, compare the relative “spread” (i.e., the distribution in the y-direction) of the data shown within each panel. A larger spread in one or more regions of the plot indicates that small changes in mechanical variable measurement could result in high error during contact point estimation. Note that a large spread does not mean there will be high error, only that there is the potential for high error.

Overall, the plots of Fig 5 show three important results. First, unsurprisingly, changes in *M*_*D*_ have a particularly large effect on *θ*_*wobj*_ and *φ*_*wobj*_. Second, the locations that have the highest potential for error occur at small values of *F*_*x*_ and *M*_*B*_, generally near the whisker tip. And third, asymmetry in the whisker shape has a large effect on mapping sensitivity when pushing the whisker concave backward.

The first column shows the effect of *M*_*D*_ on the estimated contact point. For $\frac{\partial r}{{\partial M}_{D}}$, it is clear that small changes in *M*_*D*_ have little effect on radial distance. There is some asymmetry caused by the whisker’s intrinsic curvature, with more positive values of the Jacobian element centered around *M*_*D*_ = *π* and more negative values around *M*_*D*_ = 2*π*.

The plots of $\frac{\partial \theta_{wobj}}{{\partial M}_{D}}$ and $\frac{\partial \varphi_{wobj}}{{\partial M}_{D}}$ are approximately 90-degree phase shifts of each other. These panels reflect the sudden changes in *θ*_*wobj*_ and *φ*_*wobj*_ with respect to *M*_*D*_ seen in Fig 4. Changes in *M*_*D*_ directly affect the estimates *θ*_*wobj*_ and *φ*_*wobj*_, as seen in the clear bands of purple and cyan. Because the units of *θ*_*wobj*_ and *φ*_*wobj*_ are the same, we can directly compare the two Jacobian elements. The relative magnitudes are approximately the same in the two panels, indicating that *M*_*D*_ affects the two angular variables approximately equally. Finally, we note that regions of high spread in the y-direction occur near [*π*, 2*π*] for $\frac{\partial \theta_{wobj}}{{\partial M}_{D}}$ and near $\left[\frac{\pi }{2},\frac{3\pi }{2},\frac{5\pi }{2}\right]$ for $\frac{\partial \varphi_{wobj}}{{\partial M}_{D}}$; these are regions in which the error in contact point estimation is potentially high. Notably, these regions are aligned with the zero-crossings in Fig 4, where the angular variable transitions between positive and negative. We would therefore expect these regions to have the highest spread, and thus the highest possible error, when estimating the contact point.

The second column in Fig 5 shows the effect of *M*_*B*_ on contact point estimation. Notably, all panels show a decrease in spread as *M*_*B*_ increases, indicating that the estimate is most certain at large values of *M*_*B*_. The plot for $\frac{\partial r_{wobj}}{{\partial M}_{B}}$ specifically shows that large bending magnitudes occur primarily near the proximal portion of the whisker, whereas small bending magnitudes occur near distal portions. The two panels showing the effects of *M*_*B*_ on *θ*_*wobj*_ and *φ*_*wobj*_ contain distinct regions of positive and negative angles. These regions generally correspond to negative and positive values of the Jacobian elements because the derivatives tend to have the opposite sign as the angle itself. The asymmetry in the panel for $\frac{\partial \theta_{wobj}}{{\partial M}_{B}}$ is particularly striking. To provide physical intuition for the asymmetry, recall that *θ*_*wobj*_ is the angle associated with bending the whisker either concave forwards or concave backwards. When *θ*_*wobj*_ is positive (pushing concave backwards) and *M*_*B*_ is near zero, the variable $\frac{\partial \theta_{wobj}}{{\partial M}_{B}}$ is mostly negative and the spread is large, indicating high possible error in *θ*_*wobj*_. Conversely, when *θ*_*wobj*_ is negative, $\frac{\partial \theta_{wobj}}{{\partial M}_{B}}$ is mostly positive and the spread is small, indicating a low probability of error in the estimate for *θ*_*wobj*_. Large positive deflections of *θ*_*wobj*_ are not observed for the largest magnitudes of *M*_*B*_, likely because the whisker’s intrinsic curvature causes it to slip off the object when pushed concave backwards. The angle *φ*_*wobj*_ reflects whisker bending in directions perpendicular to its intrinsic curvature, so the plot for $\frac{\partial \theta_{wobj}}{{\partial M}_{B}}$ is mostly symmetric about zero. The uncertainty is greatest near *M*_*B*_ = 0 but does not depend strongly on the sign of *φ*_*wobj*_.

The third column shows the effect of *F*_*x*_ on the estimated contact point. Just as for *M*_*B*_, spread in the data decreases as the force magnitude increases. The largest spread occurs near the whisker tip, where |*F*_*x*_| is very small. Similar to the results for *M*_*B*_, the plots of $\frac{\partial \theta_{wobj}}{{\partial F}_{X}}$ and $\frac{\partial \varphi_{wobj}}{{\partial F}_{X}}$ show a distinct separation between positive and negative angles and asymmetry in the panel for $\frac{\partial \theta_{wobj}}{{\partial F}_{X}}$.

### Reconstructing the shape of a peg from the 3D contact points

To further evaluate the quality of the mapping between (*F*_*x*_, *M*_*B*_, *M*_*D*_) and (r_wobj_, *θ*_*wobj*_, φ_*wobj*_), we performed a coordinate transformation to reconstruct the shape of the peg in the laboratory frame.

**Fig 6:**
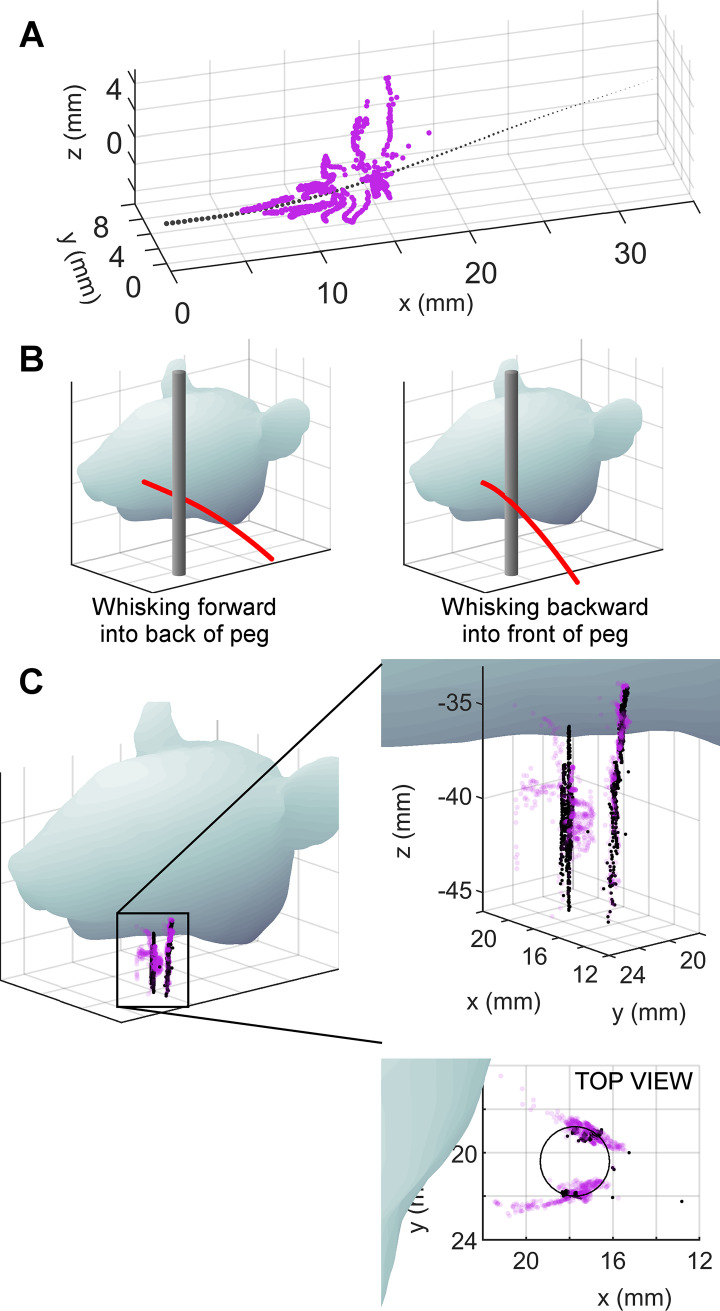
The r_wobj_, θ_wobj_, φ_wobj_ coordinates computed from the mechanical signals at the whisker base can reconstruct the shape of the peg. The data in this figure exclude small angle deflections, i.e., any point that lay less than s_closest_*tan(2°) from the whisker, where s_closest_ is the arc length of the point on the whisker closest to the contact point. **(A)** The r_wobj_, θ_wobj_, φ_wobj_ coordinates computed from the mechanical signals are plotted in whisker-centered coordinates. The whisker is the black tapered, dotted line. Each magenta dot represents a 3D contact point on the peg with respect to the whisker. Each dot is computed based on mechanical data obtained from a single video frame (1 ms resolution). These are the identical points as were plotted in Fig 4D, but are now plotted in their correct 3D spatial locations relative to the whisker. **(B)** Diagrams of the rat whisking into the peg show where reconstructed contact points are expected. The rat first whisks forward against the back of the peg, and then whisks backwards against the front of the peg. We therefore expect to see reconstructions of both the front and the back of the peg. **(C)** The same view of the rat head as in **B**, now including contact points. Tracked (“ground truth”) contact points are shown as black dots. Contact points computed from mechanical signals are shown as transparent magenta dots. Two expanded views of the reconstructed peg are shown to the right. In the side view, the points create two vertical lines: one indicating contact with the back of the peg, and the other indicating contact with the front of the peg. The top view reveals that the largest errors are found in the estimate of radial distance, rather than in estimates of the angles of contact.

Fig 6A shows the whisker in black with the predicted 3D contact points plotted in whisker-centered coordinates around the whisker. By definition [22], whisker-centered coordinates align the proximal, near-linear portion of the whisker with the x-axis, and the y-axis is set so that the whisker’s primary intrinsic curvature lies in the x-y plane. Whisker-centered coordinates can be somewhat unintuitive, so to understand this figure, imagine deflecting the whisker to each of the contact points in turn. These deflections are identical to those that occurred as a result of the rat’s active whisking against the peg. The points are dense near the proximal section of the whisker because the rat happened to whisk against the peg using the proximal portion of its whisker.

The next step was to convert these contact points to the laboratory frame, schematized in Fig 6B. Fig 6C is the identical schematic, but the peg has now been replaced with whisker-object contact points. The black dots in Fig 6C are the ground-truth contact points tracked directly from video, while the magenta dots represent the reconstruction of the peg as determined from the mappings. Notice that the “ground truth” as described here still contains tracking error; it is not the outline of the peg.

As expected, the black dots form two vertical lines: one on the back of the peg and one on the front of the peg. The reconstructed contact points are best visualized in S2 Video. The magenta dots match relatively well with the tracked peg points; however, the top view (inset) reveals that the contact point estimates are somewhat mis-matched with respect to r_wobj_. This result is consistent with the results shown in the first column in Fig 4E, depicting the goodness of fit for r_wobj_. Fig C in S1 Text gives an explanation for how errors in the peg reconstruction in both Figs 4 and 6 result from limitations in mapping resolution.

## Discussion

### Approaches towards finding the 3D whisker-object contact point

Unlike an insect antenna, a mammalian whisker has no sensors along its length: all mechanical sensing occurs at the whisker base. Therefore, a long-standing question is how an animal could determine the 3D location at which a whisker contacts an object.

Numerous studies have asked how a rodent might determine the anterior-posterior position of a peg in head-centered coordinates [5,7–20], but that is a separate problem from determining the location of an object in whisker-centered coordinates [22]. Head-centered localization schemes typically rely on information such as whisking angle, phase of the whisk, roll of the whisker, time of contact, number of contacts, or efference copy related to whisker motion. None of that information is available in the present work. The whisker-centered coordinate system used here is independent of the position and orientation of the whisker on the rat’s face and independent of the whisking cycle. The present study investigates the mechanical information sufficient for 3D location in whisker-centered coordinates and does not directly address localization in head-centered coordinates.

Previous proposed solutions for 3D localization in whisker-centered coordinates have fundamentally relied on two general approaches. The first approach involves measuring rates of change of one or more mechanical variables at the whisker base, most typically the bending moment. Specifically, it can be shown that the rate of change of bending moment is related to the radial distance of contact [5,11,24,40–44]. The second approach, used in the present work, involves combining multiple geometric variables [11] or mechanical signals [5,44] in a nonlinear manner, independent of the rates of change of these signals. This second approach is history-independent [45], because the contact point can be calculated at each instant of time. Given that primary sensory neurons of the trigeminal ganglion (Vg) are well known to exhibit both rapid and slow adaptation characteristics [17–19,39,46–59] either or both approaches are physiologically plausible.

A recent behavioral study strongly suggested that animals make at least some use of the history-independent approach [5]. In these experiments, mice whisked either against a rigid peg at a radial distance far from the whisker base or against a compliant peg at a radial distance close to the whisker base. The two peg positions were precisely chosen so as to ensure that the rate of change of bending moment was the same for both. Nonetheless, the mice could still easily distinguish between the peg locations. These results indicate that rodents do not use a localization strategy that relies exclusively on measuring the time rates of change of the bending moment.

Although the present work does not speak to whether animals use rate-based models for 3D contact point estimation, there are several arguments against such an approach. A rate-based strategy would require the animal to know whisker velocity in order to interpret the rates of change of mechanical variables, for each of its ~60 whiskers. Complicating the problem further, the velocity of a whisker at its distal end can be quite different than its velocity near the base.

The major advantage of the history-independent approach is that contact point estimation does not depend on the velocity of the whisk or on the trajectory of the whisker on the object surface. As discussed in our previous work [60,61], a history-independent approach for contact point localization would allow time-varying signals to be used concurrently for other tasks, such as detecting changes in the contact point location as the whisker slips on the object [37, 62] so as to yield estimates of object slope and curvature. Time-varying signals could also be used to detect object motion, determine compliance [5,41], or distinguish textures.

### Triplets of mechanical signals uniquely map to the 3D contact point for the biological whisker, even in the presence of dynamics and friction

Given the potential advantages of the history-independent approach, what are the mechanical variables that could be combined so as to uniquely determine the 3D whisker-object contact point? Simulation work [28] has shown that the answer depends on the shape of the whisker, including its taper and intrinsic curvature. Whiskers in the real world do not have an idealized geometry, and it is not evident that any triplets of mechanical signals would uniquely map to the contact point. In addition, the mechanical model used in the present work assumes quasistatic and frictionless conditions, both of which can affect mapping uniqueness.

The quasistatic assumption will affect the accuracy of the mappings if the dynamic response of the whisker significantly interferes with the mechanical response due to bending. For example, when a whisker initially collides with an object, it vibrates, and the quasi-static assumption is not valid. However, this interference can be avoided if the whisker maintains contact after collision long enough for dynamic effects to damp. Recent work has shown that rats maintain contact with an object for 20–50 msec [60,63], which is exactly long enough for dynamic effects to dissipate [64]. Based on these findings, we have hypothesized, that the rat deliberately retains whisker-object contact until the vibrations have damped out, so that quasistatic conditions obtain, and the spatial coordinates of contact can be estimated as the whisker sweeps along the surface before retraction [60,63,64].

Notably, in the present work, the simulated mappings were created using idealized quasistatic data, while the experimental data (run through the inverse mapping) were collected during real-world whisking behavior that necessarily included dynamic effects. Despite this mismatch, 3D contact point determination was achieved with triplets of mechanical signals to a general accuracy of a few millimeters. These results thus support our hypothesis that whisker-object contact durations are sufficient to damp vibrations and permit near-quasistatic extraction of 3D object features [60,63,64].

Friction could potentially have a large effect on mapping accuracy. In the absence of friction, each 3D contact point is associated with one and only one deflected whisker shape and one set of forces and moments at the whisker base. When friction is included, however, a single contact point location could result in multiple deflected whisker shapes and the forces and moments will depend on the history of contact. A larger coefficient of friction will have a greater effect on the mappings and could significantly reduce mapping uniqueness [5,62,65]. Frictional effects will be an important topic for future investigation.

The present work has shown that despite the non-ideal shape of the whisker, and despite real world dynamics and friction, a rodent could use triplets of mechanical information entering the follicle from a single whisker to determine the 3D contact point location in whisker-centered coordinates.

### Which combinations of mechanical variables generate unique mappings?

The present work has focused on triplets of mechanical signals because they represent the mathematically “minimum set” necessary to represent a 3D contact point. It is important to note, however, that there is no reason that the biological system should be limited to the use of these particular triplets. In addition, it is known that mechanical signals are not directly encoded by primary afferents in the follicle in a one-to-one manner. Specifically, recordings from identified afferent endings in the follicle have shown that all four of the primary afferent types (Merkel-RS, Merkel-RRC, Lanceolate, and Club-like) are broadly responsive to both bending moment as well as the axial force, although the latter two ending types appear to exhibit a stronger response to the axial force [34]. Although the precise manner in which primary afferents transform mechanical variables is still under investigation, afferent responses are clearly strongly correlated with mechanical signals at the whisker base [19,23,24,39].

First, a subset of primary sensory neurons of the trigeminal ganglion (Vg) are particularly sensitive to longitudinal deflections [58] which correspond to the axial force, *F*_*x*_. Lanceolate endings are ideally positioned to mediate this response, because their two sides are connected between the glassy membrane and the mesenchymal sheath, and they are therefore likely to respond to shearing between these two structures [34]. Correspondingly, juxtacellular recordings showed that lanceolate endings incorporate more information about the axial force and its derivative than the two types of Merkel neurons [34].

Second, it is well known that nearly all Vg neurons increase their firing rate as deflection magnitude increases [17–19,34,39,46–49,54,56,57,59,66], suggesting a sensitivity to *F*_*T*_ or *M*_*B*_. In addition, nearly all Vg neurons are strongly directionally tuned: both their firing rate as well as their adaptation characteristics depend on deflection direction [34,48–51,66]. Although all ending types in the follicle appear to be sensitive to *M*_*B*_, we suggest that Merkel-RS endings near the ring sinus are particularly well suited to respond to this signal, because they are embedded between the epithelial tissue and the glassy membrane [34].

Third, because rodents frequently touch surfaces with distal portions of their whiskers, deformation of the shaft within the follicle is likely to be dominated by the bending moment, rather than the transverse force. It will be difficult for either the rat (or a strain gage on an artificial sensor) to disambiguate the small deformation associated with *F*_*T*_ in the direction *F*_*D*_ from the much larger deformation due to *M*_*B*_ in the direction *M*_*D*_. It seems likely that these sets of signals are merged at the level of primary afferents.

With these potential coding mechanisms in mind, it is informative to examine the successful triplets for the biological whisker of the present study: (*F*_*x*_, *M*_*B*_, *M*_*D*_), (*M*_*x*_, *M*_*B*_, *M*_*D*_), and (*F*_*x*_, *F*_*D*_, *M*_*D*_). Notably, all successful mappings require *M*_*D*_, consistent with the strong directional tuning of nearly all primary afferents. In addition, all three successful triplets contain at least one signal that exhibits a gradient related to the radial distance (Fig 2). The triplet that includes *F*_*D*_ and *M*_*D*_ is surprising given that both variables encode direction information and are closely correlated. It is also intriguing that one of the successful triplets involves *M*_*X*_, as no study to date has investigated mechanisms for primary afferent encoding of this signal.

Overall, the results of the present work indicate that there is room for redundancy in the system, but that the key information for localization that must be represented is stimulus direction, magnitude, and the radial distance of contact. Provided that this information is retained, dimensionality reduction can be performed at the level of primary afferents in the whisker follicle. Such reduction would be helpful at more central levels of the trigeminal pathway, which need to integrate information about the whisker’s position and orientation on the face to localize an object in head-centered coordinates [12,17,18,22].

### Expected generalization across whiskers and whisker shapes, and to artificial whiskers

Generalizing, these results demonstrate that a rodent could use mechanical information entering the follicle from a single whisker to determine the 3D contact point location in whisker-centered coordinates. As described above, the mapping between mechanical signals and 3D geometry is history independent, so it exists independent of whisking phase or velocity, that is, the mapping remains constant for a given whisker across all whisks. In addition, the 3D contact point can be determined at all points in time during a deflection, which means that the approach can continuously solve for 3D contact location as the whisker sweeps across an object surface or even deflects against a compliant object. We note that this work is a direct and natural extension of the two-dimensional mapping from (*F*_*x*_, *M*_*B*_) to (r_wobj_, θ_wobj_) described in previous work [5,44].

We chose to focus on the first triplet because it is a direct extension of 2D work [5, 44] and because simulations have shown that it provides a unique mapping to (r_wobj_, θ_wobj_, φ_wobj_) for all tapered whiskers, regardless of whether they are straight or have intrinsic parabolic curvature [28]. Building on this work, we computed the signals (*F*_*x*_, *M*_*B*_, *M*_*D*_) at the base of the γwhisker during a bout of whisking behavior and then showed that these signals can be used to successfully estimate 3D contact point location (Fig 6 and S2 Video). In other words, the results show that the unique mappings found in simulation for an idealized whisker can extend to a real-world, non-planar whisker with non-idealized curvature.

The present study has focused on a single γ whisker, with a single set of parameters. What parameter changes affect the mappings and their uniqueness? The mappings will undoubtedly change for whiskers that have different shapes, but the question is how large these changes will be, and whether the (*F*_*x*_, *M*_*B*_, *M*_*D*_) mapping (or the other two) will retain its uniqueness.

It is reasonable to assume that almost all naturally occurring whiskers will be tapered [67–70], be largely (but not entirely) planar [25,30], and have a curvature that is mostly well-described by either a quadratic or cubic equation or an Euler spiral [25,30–32,36]. Trimming the tip of the whisker, as might occur through natural damage or barbering, would have no effect on mapping uniqueness. The only change will be that the whisker cannot reach as large a region of space. Similarly, changing Young’s modulus will scale all mechanical variables at the whisker base but not affect mapping uniqueness. Changing the radius of the whisker base, or changing the whisker arc length, will have no effect on mapping uniqueness, provided that the ratio of the base radius to tip radius remains constant and r_wobj_ is measured as a fraction of the whisker arc length (instead of in terms of absolute distance).

Changing the radius slope of the whisker (defined as the difference between the base and tip radii, divided by the arc length) will change the base-to-tip radius ratio, which will affect the mappings in highly nonlinear and often unpredictable ways. However, the mapping will remain unique for the (*F*_*x*_, *M*_*B*_, *M*_*D*_) triplet as long the whisker retains sufficient taper. If the taper is very slight, the mapping will theoretically be unique, but the resolution required to distinguish (r_wobj_, θ_wobj_, φ_wobj_) might be so high as to be impractical [28]. If the whisker is cylindrical, the mapping will be non-unique [28].

Based on these considerations, we expect unique mappings to hold for nearly all naturalistic whisker shapes. Mappings will be non-unique if there is very large in-plane curvature (e.g., if the whisker curves so much that it doubles back on itself), if there is very large out-of-plane curvature (e.g., if the whisker is a corkscrew), or if the whisker has no taper. With the exception of these cases, the problem of mapping “uniqueness” may be more accurately posed as a problem of mapping resolution. For example, Fig 4E indicates that high error occurs when r_wobj_ or θ_wobj_ are small. Future work will help resolve the origin of these errors and shed light on the extent to which the (*F*_*x*_, *M*_*B*_, *M*_*D*_) mapping will generalize across arbitrary whisker shapes.

Finally, the results of the present work pave the way to develop robots that can perform accurate 3D contour extraction [45]. Provided that the signals obtained from the base of artificial whiskers contain sufficient information about the relevant mechanics, they could be combined in a highly-nonlinear mapping to determine the 3D contact points along the contours of an object in whisker-centered coordinates. Later, these contact points can then be converted to robot-centered coordinates. Because hardware models inherently include effects such as collisions, vibrations, and friction, they may be particularly useful to help neuroscientists constrain processing at more central levels of the trigeminal system. Additionally, these models could help inform physics simulations of active whisking in real-world conditions.

## Methods

### Ethics statement

All procedures involving animals were approved in advance by the Institutional Animal Care and Use Committee (IACUC) of Northwestern University.

### Quantifying vibrissa motion and shape

We reiterate that the present study has focused on a single “gamma” (γ) whisker, with a single set of parameters, but there is no reason not to expect the results to generalize, as described in the discussion.

All whiskers on the left side of the face of a female Long-Evans rat (age ~4 months) were trimmed except for the γ whisker. The rat was gradually gentled and acclimated to body-restraint over a period of ~1 month. After acclimatization, the experiment began. During the experiment two orthogonally-mounted high speed video cameras recorded whisking behavior against a vertical peg placed in front of the animal. On each day of the experiment, the animal participated in a single 10–15 minute whisking session. The animal received a water reward for whisking against the peg. The experiment lasted approximately 2 weeks.

Two Photron 1024PCI monochrome cameras (1,000 fps, shutter speed 1/3000 second, lenses Nikon AF Micro-Nikkor 60 mm) were mounted equal distances (~60 cm) from the rat. One camera obtained a top-down (“bird’s eye”) view and the other obtained a front-on view. Each pixel (58 μm) was matched between the two cameras using a 2x2 mm^2^ checkerboard grid. In the top-down camera view, the whisker was tracked using open source software “whisk” [71]. The whisker was manually tracked in the front-on camera view. Because the two views shared the same pixel scaling and were positioned perfectly orthogonal to each other, merging was straightforward. Pixels along the x-axis were matched between the two views, and the tracked whiskers and contact points (x-y data in the top view, x-z data in the front-on view) were combined into 3D representations [72].

In each frame, the whisker basepoint position, the whisker’s angles of emergence [30, 31], and the 3D contact point location were tracked. The basepoint position and the whisker’s angles of emergence were filtered at 85 Hz to smooth tracking jitter. The cutoff 85 Hz was chosen because it both eliminated tracking noise while preserving the peaks of the whisker motion. Filtering at higher frequencies generally resulted in noisier contact point estimates; this was a gradual effect as the filtering frequency was increased. Filtering at significantly lower frequencies did not accurately capture the whisker’s bending as assessed by matching the raw tracked data with the filtered estimates.

### Generating forces from behavioral data and for mappings

The whisker shape obtained in the section “Quantifying vibrissa motion and shape” above was run through two different simulations: one to find forces and moments for the entire reachable space of the whisker, and one to obtain forces and moments at the whisker base during active whisking. The same parameters were used in both simulations. The radius at the base was 100 μm, similar to that of a C2 whisker [73], and a base radius to tip radius ratio of 15 was used [67, 70], giving the tip a radius of 6.67 μm. We assumed a typical value for Young’s modulus of 3 GPa [73] and for Poisson’s ratio, 0.38 [74].

The boundaries of the reachable space were established using two criteria: forces were applied at all locations along the whisker between 30% and 100% of its arclength, and the whisker was deflected until the simulation indicated that it had begun to “slip” (i.e., the applied force was no longer sufficient to keep the whisker in place).

In order to generate force and moment data for the mappings, we deflected the whisker to points distributed over nearly the entire space of contact points the whisker could reach. Spherical coordinates were used to describe the contact point locations. The radial distance (r_wobj_) ranged from 6 mm (30% of the whisker arc length) to 20 mm (100% of the whisker arc length) in millimeter increments. The azimuthal angle (θ_wobj_) ranged from -65° to +65° in single degree increments, and the elevation angle (φ_wobj_) ranged from -60° to +60° in single degree increments. The simulation deflected the whisker to all the resulting contact point locations. If Elastica3D could not converge to a solution for a contact point, meaning the whisker would slip off the contact point, then the point was discarded. The forces and moments for all the remaining contact points were then recorded for use in the mappings.

From the 3 seconds of data of a rat actively whisking into a peg, we used the tracked undeflected whisker shape, 3D contact point at all frames of contact, and position and orientation of the whisker at all frames of time. For each frame of contact, we used the whisker’s position and orientation to place the contact point in whisker-centered coordinates. We then used this contact point in whisker-centered coordinates as input to Elastica3D (more information available in Section 3 in S1 Text and at https://github.com/SeNSE-lab/DigitalRat) with the undeflected tracked whisker to find the forces and moments at the base of the rat whisker.

### Mapping uniqueness

The uniqueness of each of the 20 mappings using different triplet combinations of forces and moments at the whisker base was determined using the same methods described in [28]. In order to be unique, a mapping had to be found unique by both visual inspection methods and by a neural network. More details can be found in Section 4 in S1 Text.

### Sensitivity analysis

Because there are no analytic functions that define the relationship between (*M*_*D*_, *M*_*B*_, *F*_*x*_) and (*r*_*wobj*_, *θ*_*wobj*_, *φ*_*wobj*_), each element of the Jacobian (Eq 5) was evaluated using a central difference approximation while holding the other two mechanical variables constant. For each mechanical variable we chose a step size that was equal to 0.05% of each mechanical variable’s own range shown in Fig 4. For *M*_*B*_, *M*_*D*_, *F*_*x*_, this resulted in step sizes of ±3.1×10^−3^, ±6.0×10^−3^, and ±2.2×10^−4^, respectively. We used natural neighbor interpolation in MATLAB to estimate the contact point value at each step. This process resulted in 18 interpolations per point in mechanical variable space, 6 for each contact point coordinate.

Every element of the Jacobian (Eq 5) showed a large peak centered around zero and contained a large distribution of possible values. The points furthest from zero occurred at two locations. First, many were at the boundaries of the mechanical variable space, where derivatives are large likely due to interpolation error. Second, many were at points where *F*_*x*_ changed sign, which often causes a large change in contact point coordinates. To increase visual clarity in Fig 5, we found the range that contained 99% of the values for each element of the Jacobian element. This process excluded for each Jacobian element at most 1.6% of the calculated derivatives. The consequence of this approach is simply to narrow the y-axis bounds of all panels in Fig 5.

In order to avoid data overlapping, the data were split into 50 bins in each direction. Each panel of Fig 5 plots the average for each bin as a single data point. We then adjusted the transparency (alpha) value based on the number of points in that particular bin. The more transparent the point, the fewer points were in that bin. To avoid generating data points that were so transparent as to be invisible, we enforced a minimum transparency value. In each panel, any bin that had a total number of data points less than 5% of the maximum number of data points in any bin of that plot had a transparency value set to 0.05.

## Supporting information

S1 Video The 3D contact point (r_wobj_, θ_wobj_, φ_wobj_) can be determined from the signals F_x_, M_B_, M_D_ during active whisking. **(Top)** The signals F_x_, M_B_, and M_D_ vary with time as the rat whisks against the peg. The sudden change in M_D_ occurs when the rat switches from whisking forward to whisking backward against the peg. **(Middle)** Each of the three panels shows the mapping between the signals F_x_, M_B_, and M_D_ (variables on the axes) and one of the three geometric coordinates r_wobj_, θ_wobj_, and φ_wobj_ (represented with a colormap). Each mapping is a solid and is drawn as a set of semi-transparent monochromatic layers to aid visualization. In each panel, the trajectory of the black dot represents the changing values of the F_x_, M_B_, and M_D_ signals shown in the *Top* subplot of the video. At each instant of time, the color at the location of the black dot in the three panels indicates the 3D contact point coordinates. **(Bottom)** The geometric coordinates (r_wobj_, θ_wobj_, φ_wobj_) generated by the mappings shown in the *Middle* subplot.
(AVI)

S2 Video The r_wobj_, θ_wobj_, φ_wobj_ coordinates computed from the mechanical signals at the whisker base can be used to reconstruct the shape of the peg. **(Top left)** This panel shows a still image of the rat’s head, the peg location, and the tracked whisker (red). This still image can be compared with the videos in the other panels, all of which show different views of the peg “reconstruction” over time. **(Top center)** The time-varying 3D whisker-object contact point (r_wobj_, θ_wobj_, φ_wobj_) computed from the mechanical signals is plotted in whisker-centered coordinates. The whisker is the black, tapered, dotted line. Each magenta dot represents a 3D contact point on the peg with respect to the whisker. Each dot is computed based on mechanical data obtained from a single video frame (1 ms resolution). These are the identical points plotted in all other video panels, but are now plotted in their correct 3D spatial locations relative to the whisker. **(Top right)** Top-down view and front-on view of the rat as it whisks against the peg. The rat first whisks forward against the back of the peg, and then whisks backwards against the front of the peg. The whisker is traced in red for those frames in which it is in contact with the peg. **(Bottom row)** All videos in the bottom row show the gradual reconstruction of the peg’s contour based on computing contact points from the mechanical signals at the whisker base. Because the rat whisks both forward and backwards against the peg, reconstructions are observed for the peg’s front and back sides. Contact points directly tracked from the video (“ground truth”) are shown as black dots. Contact points computed from mechanical signals are shown as transparent magenta dots. **(Bottom left and center)** The left panel shows an isometric view of the rat’s head along with the time-varying whisker-peg contact points, while the center panel shows an expanded view of the contact points. Note that the contact points form two distinct vertical lines, indicating contact with the front and the back of the peg. **(Bottom right)** Reconstruction of the peg contour in top-down and front-on views, matching the videos directly above. The top-down view reveals that the largest errors are found in the estimate of radial distance, rather than in estimates of the angles of contact.
(MP4)

S1 Text Supplementary Methods. **Fig A in S1 Text. Related to Fig 1. Diagram of the overall modeling approach.** Within the gray box, the subscript “wobj” has been omitted from the geometric coordinates for brevity. **Fig B in S1 Text. Related to Fig 3. The whisker and contact point location are shown in whisker-centered coordinates. *A*:** In whisker-centered coordinates, the origin is placed at the whisker base, and the x-axis is collinear with the proximal region of the whisker. The proximal 70% of the whisker (which is close to planar) is adjusted to lie as close as possible to the x-y plane. The intrinsic curvature of the whisker points the whisker tip in the positive y-direction. ***B*:** The whisker-object (“wobj”) contact point location in whisker-centered coordinates is given by the coordinates r_wobj_, θ_wobj_, and φ_wobj_. The variable r_wobj_ is the linear distance from the origin to the contact point location. θ_wobj_, is the azimuthal angle of the contact point from the x-axis, and φ_wobj_ is the elevation angle of the contact point measured from the x-y plane. **Fig C in S1 Text. Related to Figs 4 and 5. This figure provides a step-by-step explanation for how errors in the peg reconstruction (Figs 4 and 5 of main text) result from limitations in mapping resolution.** The regions of the reconstructions with the largest error are those where F_x_ and M_B_ are small. These regions correspond to points at large radial distances or points generated by small deflections. **(A)** This subplot illustrates the identical solid as shown in Fig 3D of the main text. The solid represents the mapping of F_x_, M_B_, M_D_ to r_wobj_. A vertical plane (red) is shown cutting through the solid at M_D_ = 3π/2, and this slice of the solid is depicted in subplot (B). **(B)** This subplot shows the slice of the solid from (A) indicated by the red plane. Because the “solid” in (A) is represented by a series of monochromatic surfaces, the slice appears to be composed of different colored lines. Each line represents contact points at a single radial distance. In the lower-right hand corner of the figure, the lines are spaced far apart, indicating that the different radial distances are separated by large F_x_ and M_B_ increments. In contrast, in the upper-left corner, the lines are spaced closely together, indicating that any small error in F_x_ or M_B_ will result in a large error in r_wobj_. Note that this region includes all of the high values of r_wobj_, indicating that any contact points with a high r_wobj_ are susceptible to large errors. The inset in the bottom-left corner shows an expanded view of this region. Inset x-axis limits: [0, 5]; inset y-axis limits: [-0.25, 0.05]. **(C)** This subplot shows the same lines as subplot (B) except the color map indicates mapping error. As expected, the region where resolution demands are high have larger errors. **(D)** The same solid as in (A), but with a colormap that indicates mapping error, and a “flipped” M_B_ axis to better reveal the regions of high and low error. Layers are semi-transparent to aid with visualization. **Table A in S1 Text. Related to Fig 3. A tabulation of the uniqueness for all 20 mappings.** Boxes marked “All” indicate that the mapping is unique for the entire contact point space. Boxes marked “ELD” indicate that the mapping is unique only after excluding large deflections. Boxes marked “CF or “CB” indicate that the mapping is unique when only concave forward (CF) or concave backwards (CB) contact points are included. A mapping labeled “ELD CF” or “ELD CB” means that the mapping is unique only when concave forward or concave backwards are considered and large deflections are excluded. Some boxes include a forward slash and give multiple readings. This notation indicates that the mapping was unique for multiple conditions. For example, the way to read mapping 12 is that the mapping is unique if it is known whether the points are concave backwards or concave forward, provided large deflections are excluded. The way to read mapping 6 is that it is unique either if large deflections are excluded, or if only concave backwards points are considered. The mappings with boxes marked “not unique” had no well-defined regions of uniqueness. Two of the mappings are marked with an asterisk, indicating that they are non-unique specifically when the contact points are in the x-y plane, where M_x_ is always 0.
(PDF)
