# Supplementary material for: Demonstration of three-dimensional contact point determination and contour reconstruction during active whisking behavior of an awake rat
Source: PLoS Comput Biol. 2022 Sep 15;18(9):e1007763. doi: 10.1371/journal.pcbi.1007763 (PMC9477318; doi:10.1371/journal.pcbi.1007763)
Supplement: S1 Text — Fig A in S1 Text. Related to Fig 1. Diagram of the overall modeling approach. Within the gray box, the subscript “wobj” has been omitted from the geometric coordinates for brevity. Fig B in S1 Text. Related to Fig 3. The whisker and contact point location are shown in whisker-centered coordinates. A: In whisker-centered coordinates, the origin is placed at the whisker base, and the x-axis is collinear with the proximal region of the whisker. The proximal 70% of the whisker (which is close to planar) is adjusted to lie as close as possible to the x-y plane. The intrinsic curvature of the whisker points the whisker tip in the positive y-direction. B: The whisker-object (“wobj”) contact point location in whisker-centered coordinates is given by the coordinates rwobj, θwobj, and φwobj. The variable rwobj is the linear distance from the origin to the contact point location. θwobj, is the azimuthal angle of the contact point from the x-axis, and φwobj is the elevation angle of the contact point measured from the x-y plane. Fig C in S1 Text. Related to Figs 4 and 5. This figure provides a step-by-step explanation for how errors in the peg reconstruction (Figs 4 and 5 of main text) result from limitations in mapping resolution. The regions of the reconstructions with the largest error are those where Fx and MB are small. These regions correspond to points at large radial distances or points generated by small deflections. (A) This subplot illustrates the identical solid as shown in Fig 3D of the main text. The solid represents the mapping of Fx, MB, MD to rwobj. A vertical plane (red) is shown cutting through the solid at MD = 3π/2, and this slice of the solid is depicted in subplot (B). (B) This subplot shows the slice of the solid from (A) indicated by the red plane. Because the “solid” in (A) is represented by a series of monochromatic surfaces, the slice appears to be composed of different colored lines. Each line represents contact points at a single radial distance. I [file pcbi.1007763.s003.pdf]

Supplementary Methods

1. Overall approach

The overall approach taken in the present work is diagramed in Figure A. We first use a well-established mechanical model of whisker bending [1-4] to simulate the six components of force and moment at the base of a real rat whisker as it is deflected to all contact points it can reach. We then invert this process and create mappings from all possible triplet combinations of these six mechanical variables to the 3D contact point locations. We identify which, if any, of the mappings are associated with unique 3D contact point estimation and create lookup tables that represent these mappings. These lookup tables yield estimates of the contact point location given the forces and moments at the whisker base.

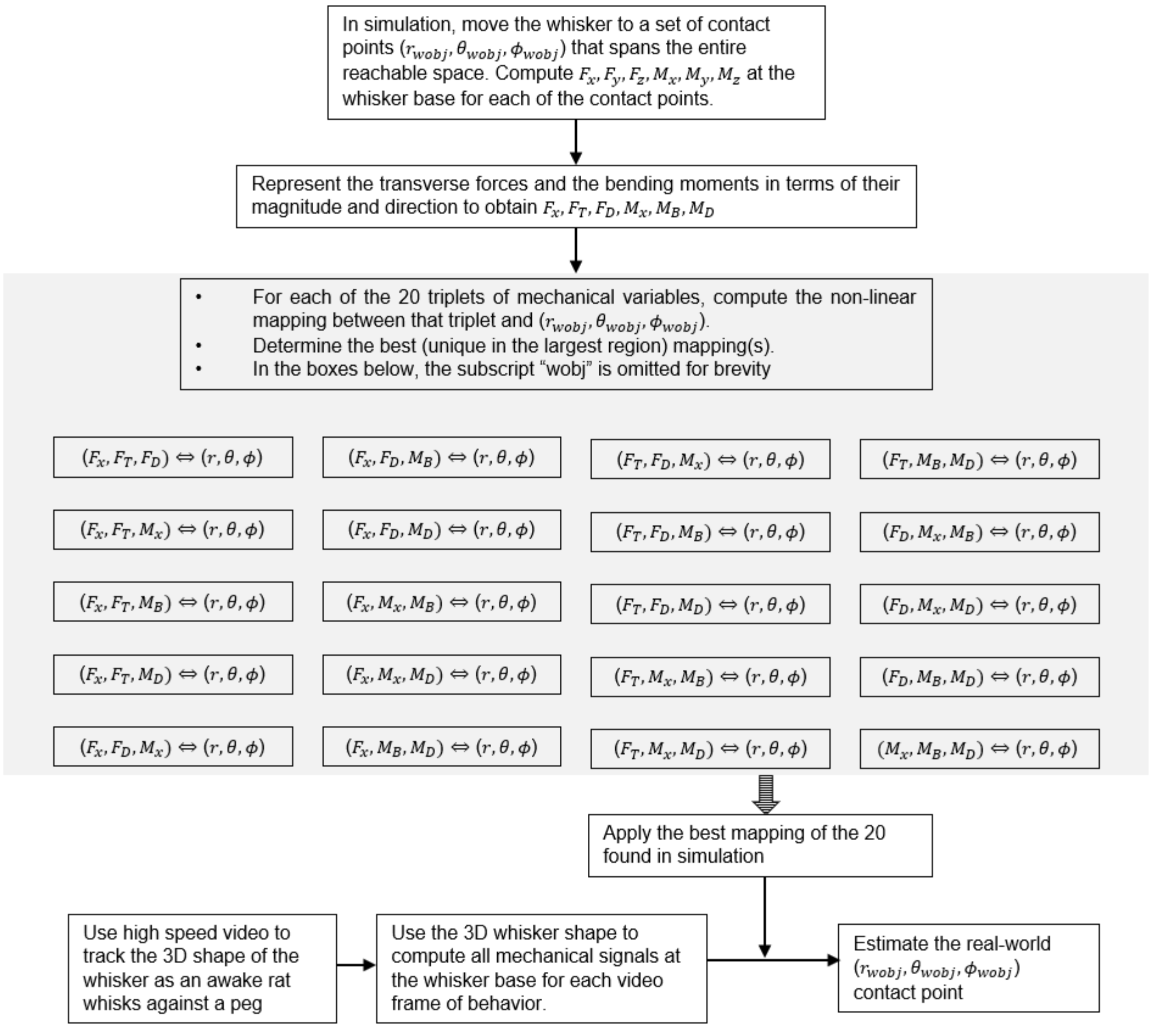

Figure A. Related to Figure 1. Diagram of the overall modeling approach. Within the gray box, the subscript "wobj" has been omitted from the geometric coordinates for brevity.

2. Finding the three-dimensional whisker shape and contact point location

As shown in panel A of Figure B, measurements of the whisker's position and orientation as well as the contact point location are made in the laboratory frame and then converted to whisker-centered coordinates [5]. The origin is located at the whisker base, and the x-axis is collinear with the proximal portion of the whisker. If the whisker is perfectly planar, it lies in the x-y plane, and the intrinsic curvature of the whisker directs the tip to point in the positive y-direction. However, most real rat whiskers are not perfectly planar. Therefore, the whisker is oriented so that the first 70% of the arc length, which is approximately planar [6-8], deviates the smallest distance from the x-y plane. The whisker is then oriented so that its tip points in the positive y-direction.

Measurement of the contact point location in whisker-centered coordinates is illustrated in panel B of Figure B. The variable r_{wobj} is the linear distance from the origin to the contact point, θ_{wobj} is the azimuthal angle of the contact point from the x-axis, and φ_{wobj} is the contact point's angle above the x-y plane.

Calculating mechanical signals at the whisker base requires a good estimate of the undeflected whisker shape, which was obtained by choosing 12 frames in which the full, undeflected whisker shape could be tracked. These frames typically occurred when the whisker was either at full protraction or full retraction without making contact, or following a duration of contact, after the whisker had detached from the peg. For these 12 frames, the whisker was tracked in each camera view, its shape fit with fifth-order polynomials, and the two views were merged to form a single 3D view and then placed in whisker-centered coordinates.

The 12 tracked whisker shapes all varied slightly from each other due to tracking error, so they were averaged together. To perform the averaging, the fifth-order whisker shapes were extended to the greatest x-distance of the longest whisker, and outlier whiskers were eliminated. The remaining whiskers were averaged in the x-y and x-z views and then merged once again to form the final "average" undeflected whisker shape.

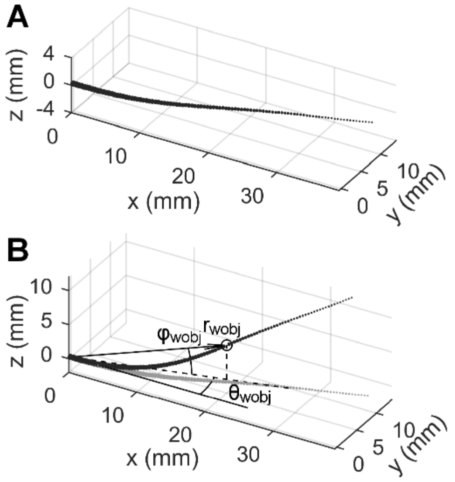

Figure B. Related to Figure 3. The whisker and contact point location are shown in whisker-centered coordinates. ***A***: In whisker-centered coordinates, the origin is placed at the whisker base, and the x-axis is collinear with the proximal region of the whisker. The proximal 70% of the whisker (which is close to planar) is adjusted to lie as close as possible to the x-y plane. The intrinsic curvature of the whisker points the whisker tip in the positive y-direction. ***B***: The whisker-object ("wobj") contact point location in whisker-centered coordinates is given by the coordinates r_{wobj}, θ_{wobj}, and Φ_{wobj}. The variable r_{wobj} is the linear distance from the origin to the contact point location. θ_{wobj}, is the azimuthal angle of the contact point from the x-axis, and Φ_{wobj} is the elevation angle of the contact point measured from the x-y plane.

3. Background on the whisker bending model, Elastica3D

Forces and moments at the base of the whisker for the full range of contact points were computed using a well-established mechanical model, which we call "Elastica3D" [1-4], found at https://github.com/SeNSE-lab/DigitalRat. The physical parameters used were based on typical whisker values: 100 μm base diameter and a base-to-tip radius ratio of 15 [6, 9, 10], yielding a tip diameter of 6.67 μm. Young's modulus was set to 3 GPa [11] and the shear modulus G was obtained by inserting the value of Poisson's ratio for keratin, 0.38 [12], into the following equation:

$$G = \frac{E}{2(1+\upsilon)} \qquad \text{(eq. 1)}$$

The model, Elastica3D, has already been described in detail in previous studies [1-4], but we reiterate the main principles here. The model takes as input the undeflected shape of the whisker and a contact point location. It then deflects the whisker by applying a point load of magnitude $F_{applied}$ at an arc length $S_{applied}$ from the base and at an angle $z_{applied}$ about the whisker's axis. As described in the following paragraphs, Elastica3D runs an optimization over these three variables until the location of the load on the deflected whisker is coincident with the specified contact point location.

For each iteration of the optimization, Elastica3D applies a point force described by $F_{applied}$, $s_{applied}$, and $z_{applied}$ to the whisker. According to quasistatic limits, the applied force remains perpendicular to the whisker's axis at the point of contact.

This force generates a bending moment, M_B, and a torque, M_X, at each node, and each node rotates in two directions by amounts dictated by linear elastic beam theory. The node rotates about an axis perpendicular to the distal link's axis by an amount described by the following equation:

$$d\theta = ds\left(\frac{M_B}{EI}\right) \qquad \text{(eq. 2)}$$

where ds is the length of the link, E is the Young's modulus, and I is the area moment of inertia calculated by $I = \pi r^4/4$, where r is the radius of the whisker at that node.
The node also rotates about axis defined by the distal link by the amount described in the following equation:

$$d\varphi = ds\left(\frac{M_X}{GJ}\right) \qquad \text{(eq. 3)}$$

where ds is again the length of the link, and J is the polar moment of inertia calculated by $J = \pi r^4/2$, and r is the radius of the whisker at that node.

We also impose a rigid boundary condition at the base, meaning that the basepoint does not translate and the proximal-most link does not rotate.

The optimization algorithm used in Elastica3D is the Nelder-Mead algorithm, provided by the fminsearch function in MATLAB, and the cost function of the optimization is the Euclidean distance from the location of the point force on the deflected whisker to the user-specified contact point location. When this distance becomes zero, Elastica3D has found a solution for the whisker and contact point location and outputs both the deflected shape of the whisker and the resultant forces and moments at the whisker base. If the optimization cannot find a solution where the output of the cost function is zero, then Elastica3D determines that the whisker cannot reach the input contact point and declares that the whisker has "slipped off" the contact.

4. Methods for determining mapping uniqueness

Mapping uniqueness was determined both through visual inspection and neural network analysis. As depicted in Figure 3 of the main text, the mappings can be visualized as a series of monochromatic surfaces; the colors of the surfaces represent the value of r_{wobj}, θ_{wobj}, or Φ_{wobj} for a given combination of mechanical variables. If any of these monochromatic surfaces overlap, then the mapping is not unique: the overlap implies that a single reading of the three mechanical variables used would give two or more conflicting results for the contact point location. Thus, in order to pass the visual inspection for uniqueness, a mapping could not have any apparent overlap.

The second uniqueness validation involved using a neural network as a function approximator. In this approach, a neural network was constructed to solve for the highly nonlinear function that related the three selected mechanical variables to the contact point, r_{wobj}, θ_{wobj}, and Φ_{wobj}. If the neural network could not solve for the nonlinear function within certain limitations (described in detail below), then the mapping was determined to be non-unique.

We used the MATLAB built-in neural network functionality to create, train, and run the neural networks. All networks used the same architecture, selected to solve best for highly nonlinear functions. All networks had three input nodes for the three mechanical signals, three output nodes for the three whisker-object contact point coordinates, and two hidden layers of ten nodes each. The nodes in the hidden layer used a hyperbolic tangent sigmoid function, and the nodes in the output layer used a linear transfer function. The networks were trained using Levenberg-Marquardt backpropagation, and the performance function was the mean squared normalized error. The training set encompassed 35% of the data. MATLAB's built-in training functions used 7.5% of the remaining data for testing during training and a further 7.5% for training validation. The remaining 50% of the data was used to calculate errors used for uniqueness determination.

The neural network error was defined as the difference between the three variables r_{wobj}, θ_{wobj}, and Φ_{wobj} and those same three variables as output by the neural network. These errors were then weighted by $r_{wobj}^2 cos\ (\phi_{wobj})$ to determine the median errors in r_{wobj}, θ_{wobj}, and Φ_{wobj}. This weighting was required to ensure that the contact points, which were equally distributed in spherical coordinates, had equal weight in Cartesian coordinates. Mappings were considered unique only if the median weighted neural network errors were less than or equal to 1.5 mm for r_{wobj}, and less than or equal to 1 degree for θ_{wobj} and Φ_{wobj}.

We found that some mappings were unique only in select regions of the space. For example, some mappings were unique only when points that involved large deflections were excluded. Contact points were determined to be large deflection if the link that rested on the contact point was oriented in the negative x-direction. Other mappings were unique only when points that resulted from either "concave forward" or "concave backwards" collisions were excluded. Points were determined to be concave forward if, in the top-down view in whisker-centered coordinates, they lay below (in the negative y-direction) the points that defined the curve of the whisker. Points above the whisker (in the positive y-direction) were considered concave forward.

Finally, it is important to note a subtlety in the term "uniqueness." Neural network errors – averaged across the entire mapping – could be well below threshold for all three geometric coordinates, but still have tiny regions of extreme non-uniqueness. This situation occurred for the case of the mapping used throughout this study (F_X, M_B, M_D), resulting in less accurate r_{wobj} estimates for points very near the whisker, as further discussed in the text for Figure 5 and 6.

5. Obtaining estimates of mapping error

As described in the previous section, estimates of mapping uniqueness were based on computing the "neural network error." The neural network error is a measure of the performance of the neural network, i.e., its ability to predict 50% of the data (the test set) based on the other 50%.

However, errors between actual contact point and predicted contact point are present throughout the entire mapping, and thus a different metric is required to quantify the error and resolution of the mapping. The "mapping error" is found using 100% of non-normalized data.

To find the mapping error, all combinations of F_X, M_B, and M_D from the entire range of whisker-object contact points were input into the neural network, which calculated contact point locations based on these mechanical signals. The contact point locations estimated by the neural network were then compared to the actual contact point locations, and the Euclidean distance between these two defined the mapping error at each contact point.

6. Imperfections in peg reconstruction and effects of resolution

Figures 4, 5, and 6 of the main text demonstrate that the mapping based on F_X, M_B, and M_D can be used to determine the 3D whisker-object contact point location and thus ultimately the contour of the peg during active whisking behavior of the awake rat. However, these two figures also show a number of points that stray from the actual contact points, particularly in the r_{wobj} dimension.

These imperfections originate from limitations in mapping resolution, as explained step-by-step in Figure C. Panel A of Figure C begins by showing the visualization of the mapping to r_{wobj}, replicated from Figure 3D of the main text. A vertical plane is shown bisecting the mapping "solid" at $M_D = 3\pi/2$, where contact points are near the x-y plane. Panel B of Figure C shows the cross-section of the r_{wobj} mapping at this plane. The cross-section consists of a series of curved lines because the solid in panel A is drawn as a series of monochromatic surfaces, and the cross-sectional cut passing through these surfaces generates lines.

As described in the main text, the contact points with the largest errors were those very close to the whisker, associated with small deflections. In Panel B of Figure C, these contact points are most clearly observed in the inset, which focuses on small values of F_X and M_B. Notice that in this region, the different monochromatic surfaces are layered very close to each other, and – given that each surface represents a different r_{wobj} for contact points – it is clear that the resolution requirements in this area are very demanding. During small deflections, even tiny errors in either F_X or M_B will cause large error in r_{wobj}. This requirement for high resolution contrasts with the regions of large F_X and M_B vales in the lower right-hand corner of panel B of Figure C, where the layers are spaced far apart.

Panel C of Figure C confirms that the inset region of panel B of Figure C is indeed where high errors occur. Panel C is identical to that shown in panel B, but the colormap for the surfaces now represent the "mapping error," as described in the previous section. This error is calculated by taking the mechanical signals originally obtained when generating data from the mappings and running them through the neural network to produce r_{wobj}, θ_{wobj}, and Φ_{wobj} values. If the neural network worked perfectly, these values would exactly match the points used to generate the mappings. Consistent with expectations, the largest errors occur in regions with small deflections, where the layers are close together. Finally, panel D of Figure C shows the same surface as panel A of Figure C, but with a colormap indicating mapping error and a flipped M_B axis. This subplot shows even more clearly that the errors are highest when deflections are small.

In future work, this error could potentially be reduced by optimizing the neural network architecture and associated training algorithm, or perhaps by using a larger training dataset. An alternative possibility is that the large error in the region of small F_X and M_B might actually reflect non-uniqueness at the resolution analyzed. Although previous work has demonstrated that a perfectly parabolic curve produces a unique mapping with the triplet F_x, M_B, and M_D, the real whisker used in the present work curves out of the x-y plane, and has irregular curvature.

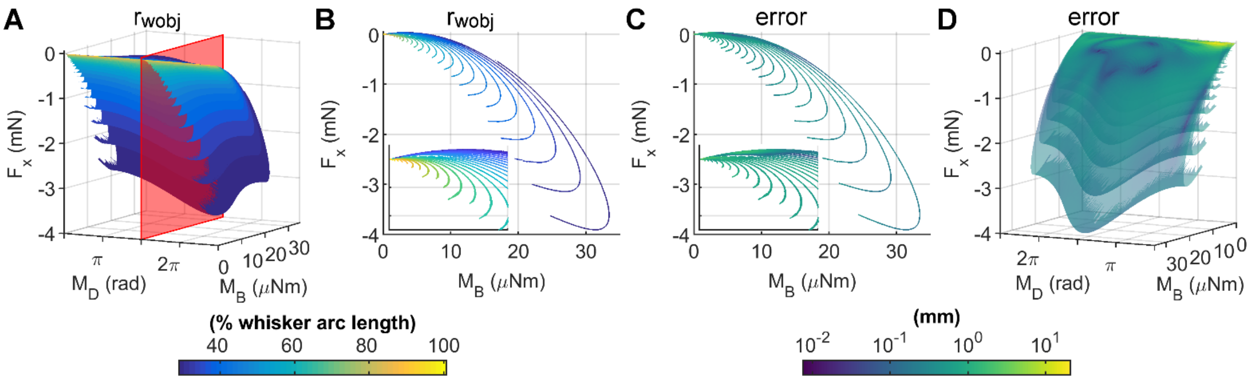

Figure C. Related to Figures 4, 5 and 6. This figure provides a step-by-step explanation for how errors in the peg reconstruction (Figures 4-6of main text) result from limitations in mapping resolution. The regions of the reconstructions with the largest error are those where F_X and M_B are small. These regions correspond to points at large radial distances or points generated by small deflections. **(A)** This subplot illustrates the identical solid as shown in Figure 3D of the main text. The solid represents the mapping of F_X, M_B, M_D to r_{wobj}. A vertical plane (red) is shown cutting through the solid at $M_D = 3\pi/2$, and this slice of the solid is depicted in subplot (B). **(B)** This subplot shows the slice of the solid from (A) indicated by the red plane. Because the “solid” in (A) is represented by a series of monochromatic surfaces, the slice appears to be composed of different colored lines. Each line represents contact points at a single radial distance. In the lower-right hand corner of the figure, the lines are spaced far apart, indicating that the different radial distances are separated by large F_X and M_B increments. In contrast, in the upper-left corner, the lines are spaced closely together, indicating that any small error in F_X or M_B will result in a large error in r_{wobj}. Note that this region includes all of the high values of r_{wobj}, indicating that any contact points with a high r_{wobj} are susceptible to large errors. The inset in the bottom-left corner shows an expanded view of this region. Inset x-axis limits: [0, 5]; inset y-axis limits: [-0.25, 0.05]. **(C)** This subplot shows the same lines as subplot (B) except the color map indicates mapping error. As expected, the region where resolution demands are high have larger errors. **(D)**The same solid as in (A), but with a colormap that indicates mapping error, and a “flipped” M_B axis to better reveal the regions of high and low error. Layers are semi-transparent to aid with visualization.

6. Mappings using other mechanical variable triplets

We also evaluated uniqueness for the remaining 19 mappings resulting from all the different triplet combinations of the six mechanical variables at the whisker base. The uniqueness results are listed in Table A (next page). Each mapping triplet is listed in the columns on the left, and the rightmost column describes the region of the contact point space in which the mapping is unique. Mappings that are labeled “All” are unique when all contact points are included. Note that the F_x, M_B, M_D mapping featured in the present work is one of three possible combinations that yields a unique mapping for all contact points.

Some mappings are labeled “ELD,” indicating that the mappings are unique only when large deflections are excluded. Large deflections are defined as those that deflect the whisker past the point where F_D experiences a 180° flip.

Mappings can also be labeled “CF” or “CB,” meaning that the mappings are unique only when either concave forward or concave backward collisions are considered, respectively.

A mapping labeled either “ELD CF” or “ELD CB” means that the mapping is unique when only concave forward or concave backwards collisions are considered and large deflections are excluded.

Only two mappings, on rows 19 and 20, were not unique under any of these conditions.

Some mappings are unique under multiple conditions, such as the F_T, F_D, M_D mapping on row 6. It is unique either if large deflections are excluded or if only concave backward collisions are included. To represent this, the right-hand column in Table A (next page) is split into multiple boxes to show both “ELD” and “CB.”

Mappings can also be marked with a single asterisk. This asterisk indicates that although the mapping is unique for the regions indicated, it is non-unique in the x-y plane. The mapping cannot discriminate between points in this region because M_X is always equal or nearly equal to 0.

Although the bulk of this study has focused on the F_X, M_B, M_D mapping, this table indicates alternative mapping combinations that can be further explored in future studies.

Table A. Related to Figure 3. A tabulation of the uniqueness for all 20 mappings. Boxes marked “All” indicate that the mapping is unique for the entire contact point space. Boxes marked “ELD” indicate that the mapping is unique only after excluding large deflections. Boxes marked “CF or “CB” indicate that the mapping is unique when only concave forward (CF) or concave backwards (CB) contact points are included. A mapping labeled “ELD CF” or “ELD CB” means that the mapping is unique only when concave forward or concave backwards are considered and large deflections are excluded. Some boxes include a forward slash and give multiple readings. This notation indicates that the mapping was unique for multiple conditions. For example, the way to read mapping 12 is that the mapping is unique if it is known whether the points are concave backwards or concave forward, provided large deflections are excluded. The way to read mapping 6 is that it is unique either if large deflections are excluded, or if only concave backwards points are considered. The mappings with boxes marked “not unique” had no well-defined regions of uniqueness. Two of the mappings are marked with an asterisk, indicating that they are non-unique specifically when the contact points are in the x-y plane, where M_X is always 0.

	**Inputs**			**When is the mapping unique?**
	**1**	**2**	**3**	
**1**	**FX**	**MB**	**MD**	All
**2**	**MX**	**MB**	**MD**	All
**3**	**FX**	**FD**	**MD**	All
**4**	**FX**	**FT**	**MD**	ELD
**5**	**MB**	**FT**	**MD**	ELD
**6**	**FT**	**FD**	**MD**	ELD / CB
**7**	**MX**	**FD**	**MD**	CB
**8**	**MX**	**FX**	**MB**	CB
**9**	**MX**	**FX**	**MD**	CF* / CB
**10**	**MB**	**FD**	**MD**	ELD CF / CB
**11**	**MX**	**FX**	**FT**	ELD CF / ELD CB
**12**	**MX**	**FT**	**MB**	ELD CF / ELD CB
**13**	**MX**	**FT**	**MD**	ELD CF* / ELD CB
**14**	**MX**	**MB**	**FD**	ELD CF
**15**	**MX**	**FT**	**FD**	ELD CF
**16**	**FX**	**MB**	**FD**	ELD CB
**17**	**FT**	**MB**	**FD**	ELD CB
**18**	**FX**	**FT**	**FD**	ELD CB
**19**	**FX**	**MB**	**FT**	Not unique
**20**	**FX**	**MX**	**FD**	Not unique

References

1. Huet, L.A. and M.J.Z. Hartmann, *Simulations of a Vibrissa Slipping along a Straight Edge and an Analysis of Frictional Effects during Whisking.* Ieee Transactions on Haptics, 2016. **9**(2): p. 158-169.
2. Huet, L.A., J.W. Rudnicki, and M.J.Z. Hartmann, *Tactile sensing with whiskers of various shapes: Determining the three-dimensional location of object contact based on mechanical signals at the whisker base.* Soft Robotics, 2017. **4**(2): p. 88-102.
3. Huet, L.A., C.L. Schroeder, and M.J.Z. Hartmann, *Tactile signals transmitted by the vibrissa during active whisking behavior.* Journal of Neurophysiology, 2015. **113**(10): p. 3511-3518.
4. Yang, A.E.T. and M.J.Z. Hartmann, *Whisking kinematics enables object localization in head-centered coordinates based on tactile information from a single vibrissa.* Frontiers in Behavioral Neuroscience, 2016. **10**: p. 145.
5. Bush, N.E., S.A. Solla, and M.J.Z. Hartmann, *Whisking mechanics and active sensing.* Current Opinion in Neurobiology, 2016. **40**: p. 178-188.
6. Belli, H.M., et al., *Quantifying the three-dimensional facial morphology of the laboratory rat with a focus on the vibrissae.* PLoS ONE, 2018. **13**(4): p. e0194981.
7. Knutsen, P.M., A. Biess, and E. Ahissar, *Vibrissal kinematics in 3D: Tight coupling of azimuth, elevation, and torsion across different whisking modes.* Neuron, 2008. **59**(1): p. 35-42.
8. Towal, R.B., et al., *The morphology of the rat vibrissal array: a model for quantifying spatiotemporal patterns of whisker-object contact.* PLoS Computational Biology, 2011. **7**(4).
9. Hires, S.A., et al., *Tapered whiskers are required for active tactile sensation.* eLife, 2013. **2**: p. e01350.
10. Williams, C.M. and E.M. Kramer, *The advantages of a tapered whisker.* PLOS ONE, 2010. **5**(1): p. e8806.
11. Quist, B.W., R.A. Faruqi, and M.J.Z. Hartmann, *Variation in Young's modulus along the length of a rat vibrissa.* Journal of Biomechanics, 2011. **44**(16): p. 2775-2781.
12. Etnier, S.A., *Twisting and bending of biological beams: Distribution of biological beams in a stiffness mechanospace.* The Biological Bulletin, 2003. **205**(1): p. 36-46.
